# Supplementary material for: Protocol for a 2-year longitudinal study of eating disturbances, mental health problems and overuse injuries in rock climbers (CLIMB)
Source: BMJ Open. 2023 Sep 13;13(9):e074631. doi: 10.1136/bmjopen-2023-074631 (PMC10503345; doi:10.1136/bmjopen-2023-074631)
Supplement: Author's manuscript [file bmjopen-2023-074631.draft_revisions.pdf]

BMJ Open

BMJ Open is committed to open peer review. As part of this commitment we make the peer review history of every article we publish publicly available.

When an article is published we post the peer reviewers' comments and the authors' responses online. We also post the versions of the paper that were used during peer review. These are the versions that the peer review comments apply to.

The versions of the paper that follow are the versions that were submitted during the peer review process. They are not the versions of record or the final published versions. They should not be cited or distributed as the published version of this manuscript.

BMJ Open is an open access journal and the full, final, typeset and author-corrected version of record of the manuscript is available on our site with no access controls, subscription charges or pay-per-view fees (http://bmjopen.bmj.com).

If you have any questions on BMJ Open's open peer review process please email info.bmjopen@bmj.com

BMJ Open

CLIMB:Climbers' Longitudinal attitudes towards Injuries, Mental health, and Body image: Protocol for a two-year longitudinal study of risk and prognostic factors for mental- and physical health problems among climbers.

Journal:	*BMJ Open*
Manuscript ID	bmjopen-2023-074631
Article Type:	Protocol
Date Submitted by the Author:	12-Apr-2023
Complete List of Authors:	Edlund, Klara; Sophiahemmet University, Department of Health Promotion Science; Karolinska Institute, Unit of Intervention Research on Worker Health, Institute of Environmental Medicine<br>Nigicser, Isabel; Sahlgrenska Academy, Ortopedi<br>Sansone, Mikael; Sahlgrenska Academy, Ortopedi<br>Identeg, Fredrik; Sahlgrenska Academy<br>Hedelin, Henrik; Sahlgrenska University Hospital,<br>Forsberg, Niklas; N/A<br>Tranaeus, Ulrika; Swedish School of Sport and Health Sciences, Department of FNB; Karolinska Institute, Institute of Environmental Medicine
Keywords:	Eating disorders < PSYCHIATRY, MENTAL HEALTH, SPORTS MEDICINE

SCHOLARONE™
Manuscripts

CLIMB:
Climbers' Longitudinal attitudes towards Injuries, Mental health, and Body image: Protocol for a two-year longitudinal study of risk and prognostic factors for mental- and physical health problems among climbers.

Klara Edlund[1,2]
Isabel Nigicser[3], ORCID: 0000-0002-0707-7276
Mikael Sansone[3], ORCID: 0000-0002-9844-1140
Fredrik Identeg[3], 0000-0002-7295-9865
Henrik Hedelin[3], ORCID: 0000-0002-0657-2232
Niklas Forsberg, ORCID: 0000-0002-5151-7144
Ulrika Tranaeus[4], ORCID: 0000-0002-2102-6352

Correspondence: Klara Edlund, (ORCID: 0000-0002-2614-5174)

Division of Psychology, Department of Clinical Neuroscience, Karolinska Institutet, SE-171 77, Stockholm, Sweden. E-mail: Klara.edlund@ki.se, phone: +46-706947430

Affiliations

1) Division of Psychology, Department of Clinical Neuroscience, Karolinska Institutet, SE-171 77, Stockholm, Sweden.

2) Musculoskeletal and Sports Injury Epidemiology Center, Department of Health Promotion Sciences, Sophiahemmet University, Stockholm, Sweden.

3) Department of Orthopedics, Institute of Clinical Sciences, Sahlgrenska Academy, University of Gothenburg, Gothenburg, Sweden.

4) Department of Physiology, Nutrition, Biomechanics; Sport Performance & Exercise Research & Innovation Center - Stockholm, SPERIC-S; The Swedish School of Sport and Health Sciences, Stockholm Sweden

For peer review only - http://bmjopen.bmj.com/site/about/guidelines.xhtml

ABSTRACT

Introduction:
The project will follow Swedish competitive climbers over a two-year period regarding disturbed eating (DE), mental health status, overuse injuries, bone health, and symptoms indicative of relative energy deficiency (RED-S).

Method and analysis:
This prospective longitudinal study aims to recruit the whole eligible population of Swedish competitive climbers. Data will be collected using web-based questionnaires, four times over two years as well as bone health tests (Dual-Energy X-ray Absorptiometry, DEXA). Statistical analyses will include multinominal logistic regression, multivariate analysis of variance MANOVA and Structural equation modelling (SEM).

Ethics and dissemination:
The study was approved by the Swedish ethics authority (2021-05557-01). Results will be disseminated through peer-reviewed research papers, research conferences, and stakeholder communications.

Trial registration number: NCT05587270.

Strength and limitations of this study:

- The study will increase the understanding of the aetiology of disturbed eating, mental health status, overuse injuries, bone health and symptoms indicative of relative energy deficiency in elite and advanced climbers.
- Climbers will be studied over a period of two years and will be compared to normal controls.
- The use of valid and reliable instruments and measurement methods will improve the internal validity of the estimated associations.
- The use of DEXA complements the self-report data with objective measurements.
- One limitation is the use of self-report questionnaires which yield subjective data and increase the risk of attrition.

Key words: Rock climbing, disordered eating, relative energy deficiency, DEXA, injuries, mental health problems, bone health.

PUBLIC SIGNIFICANCE STATEMENT

Climbing has rapidly grown in popularity as can be seen by the inclusion of the sport in the 2020 Olympic games. Climbing is a weight sensitive sport where performance is enhanced by low bodyweight relative to strength. Due to frequent reports of health conditions in athletes, along with athletes being reluctant to seek professional help we emphasize the need for specific knowledge regarding climbers.

INTRODUCTION

For peer review only - http://bmjopen.bmj.com/site/about/guidelines.xhtml

For peer review only

Rock climbing has in recent years gained increased popularity as a recreational and competitive sport, as reflected by a rapid increase of practitioners, media coverage, and the recent inclusion of the sport in the Olympics of 2020 and 2024. Research on climbing related Disordered Eating (DE) and Relative Energy Deficiency in Sports (RED-S) is sparse. The recent increase in the numbers of climbers, both competing athletes and recreational climbers, have brought attention to health issues related to climbing. Climbing as a sport places high demands on strength, mobility, and endurance of the athlete. Especially for high performance climbing, relative strength to weight ratio has been shown to be of importance for performance resulting in lean body shapes, commonly with low BMIs, fat percentages and low calory intakes [1]. A recent study by Joubert et al [2] examined 114 female climbers competing at the World Cup level and found that 15.8% presented with current amenorrhea. Additionally, among these climbers with amenorrhea, a larger percent revealed having struggled with DE compared to those without menstrual disturbances (13.5% vs 22.5%, respectively). Studies with a more comprehensive examination of the etiology of RED-S, performance, and injuries among climbers has been called for.

Disordered eating is characterized by a deep dissatisfaction with one's own body and/or shape, and is associated with life-threatening medical and psychiatric comorbidities [3].The prevalence of clinical and sub-clinical eating disorders (EDs) in elite athletes have been reported to be 13-16% higher than for the general population [4-6]. In sports where the importance of low body weight is considerable, athletes who compete appear to be particularly vulnerable to the development of disturbed eating patterns [7]. This includes sports such as ballet, gymnastics, and figure-skating [8]. Even at the non-professional level, performers of lean sports emphasizing thinness and muscularity such as body-building and ballet exhibit disordered eating behaviors [9]. Suggested sport-specific, risk factors for eating disturbances include frequent weight regulation, dieting and experienced pressure to lose weight [10, 11]. The International Federation of Sport Climbing (IFSC) has recently implemented a lower weight limit of BMI ≥ 17.5 for eligibility to compete in international competition since low body weight (BMI < 17.5) has repeatedly been observed among competitors. To the same end, the Austrian Sport Climbing Organization has set a lower age limit for competitors: 18 years for males, and 17 years for females to mitigate the risk of the developing eating disturbances [12].

RED-S is a syndrome referring to impaired physiological functioning caused by relative energy deficiency and includes, but is not limited to, impairments of metabolic rate, menstrual function, bone health, immunity, protein synthesis and cardiovascular health. Low energy availability (LEA) appears to be an important variable, as studies report that LEA impairs reproductive function and bone formation. As well as decreasing bone formation, it has also been seen to induce a higher increase bone resorption in women compared to men [13].

Compulsive exercise (CE) consists of maladaptive compensatory behaviors and serves as a strategy for emotion regulation – often closely linked to eating disturbances. It is characterized by an inability or unwillingness to cut down or stop the behavior despite adverse health consequences. Furthermore, exercising for regulation of negative emotion has been consistently identified as contributory factor to the development and maintenance of EDs [14]. This contribution does not lie specifically in the presence of negative emotion, but rather in the compulsive exercise habits that develop as an outlet for these emotions, which in turn is a risk factor for developing disturbed eating.

For peer review only - http://bmjopen.bmj.com/site/about/guidelines.xhtml

Body dissatisfaction is associated with drive for thinness [15, 16], dieting [17], and disordered eating patterns [17, 18]. The exposure to physical ideals has been seen to have a stronger immediate impact on girls than boys [19], and preoccupation with weight, maladaptive methods for weight control, and body dissatisfaction are known precursors of more serious clinical eating disorders, both among athletes and in the general population [20] [10, 11]. However, athletes from leanness-focused sports report higher rates of eating disturbances compared to athletes from non-weight sensitive sports[8].

In 2020, one of the first papers on eating disorders and climbing ability was published, which assessed 604 climbers (ages 32 ± 9 years). It presented demographic data according to the International Rock-Climbing Research Association's (IRCRA) directives [21]. The results showed that higher level climbers had significantly lower BMI among both genders, and that 8.6% of the climbers reported disordered eating behaviors. Additionally, 4.2% reported having been treated for an eating disorder and there was an increased risk for eating disorders among female elite climbers compared to males [22].

CLIMB, an acronym for our study *C*limber's *L*ongitudinal attitudes towards *I*njury, *M*ental health, and *B*ody Image, will be of hypothesis-testing nature. Our main hypothesis is that elite and sub-elite[1] climbers will present more DE symptoms compared with controls, with elite climbers showing more symptoms than sub-elite climbers. A secondary hypothesis is that climbers who report more eating disturbances will present with higher levels of health problems including decreased bone density, compulsive exercise, depression, body dissatisfaction, menstrual disturbances and more overuse injuries as compared to climbers with less symptoms of eating disturbances, as these secondary variables are correlates of EDs. We expect these patterns to be stable over time. The authors will also examine the gender differences in these categories and expect, for example, to find higher percentages of DE among women than men, following the pattern of the presented literature. Due to the rapidly increasing popularity of recreational and competitive climbing, longitudinal cohort studies are urgently needed to understand the etiology of health problems related to eating disorders in climbers in particular. Studies that identify risk- and prognostic factors are required to develop health promotion and disease prevention strategies, which ultimately can reduce the burden of disease in climbers worldwide.

METHOD AND ANALYSIS

Study design and participants

CLIMB will be executed as a prospective longitudinal study, using web-based questionnaires with four measures over a two-year period (baseline, and 6-, 12- and 24 months follow-up) and additional follow-ups at 3 and 5 years after baseline. The whole eligible population of Swedish climbers on elite- or sub-elite levels according to the IRCRA will be invited to participate in the study. Information about the study will be distributed by the Swedish Climbing Federation (https://www.klatterforbundet.se) via climbing gyms and national teams. Information about the study will also be promoted on social media channels such as Facebook and Instagram. Participants will be both male and female, 13 years and older.

[1] The authors use the terms "sub-elite" and "advanced" interchangeably. The term "advanced" is derived from the IRCRA classifications, and "sub-elite" is more colloquially understood in this context. Both terms, however, refer to the same level of climbing.

For peer review only - http://bmjopen.bmj.com/site/about/guidelines.xhtml

The inclusion criteria for the climbing group follow the grade classifications of IRCRA [21] . Each climber must climb at a minimum of advanced level. For women, this means having climbed a boulder route rated harder than 6B or a lead route rated 6c within the last year. For men, this means having climbed a boulder route rated harder than 6B or a lead route rated 7a+ within the last year.

A non-athlete control group (n=180) from the general population, matched in age and gender, will be invited to participate in the study. They will be recruited via social media and universities in Gothenburg and Stockholm. The control group's eligibility will be established by answering a two-question survey regarding general physical activity (detailed in the Instruments section below), as well as being above 13 years of age. An exclusion factor, for example, would be for a control participant to have trained and competed in any sport at the elite level prior to answering the questionnaire.

Instruments

The first portion of the questionnaire aims to confirm that participants are eligible for inclusion, with a climbing level of advanced or above as classified by IRCRA. Demographic data will be gathered and reported including factors such as age, time practicing the sport, amount of weekly training hours, climbing level, and gender. Symptoms related to eating disturbances, RED-S, body image, compulsive training, perfectionism, mental health problems, poor sleep quality, overuse injuries, and menstrual dysfunction will thereafter be measured (see table 1 below).

In addition to the web-based questionnaire, bone health using Dual-Energy X-ray Absorptiometry (DEXA) will be measured for climbing participants at baseline.

Table 1

Variable	Instrument	Baseline	Follow-up
Eating Disorder Symptoms	The Eating Disorders Questionnaire (EDE-Q 6.0; Fairburn & Beglin, 1994)	X	X
Overuse Injuries	The Oslo Sports Trauma Research Center Overuse Injury Questionnaire (OSTRC-O; Clarsen et al., 2013)	X	X
Perfectionism	The Frost Multidimensional Perfectionism Scale (FMPS; Frost, Marten, Lahart & Rosenblate, 1990)	X	X
Compulsive Training	The Compulsive Exercise Test (CET; Taranis, Touyz & Meyer, 2011)	X	X
Body Dissatisfaction	Body Shape Questionnaire, brief version (BSQ-8C; Welch, Lagerström & Ghaderi, 2012)	X	X
Mental Health	Depression Anxiety Stress Scale-21 (DASS-21; Lovibond & Lovibond, 1995)	X	X
Sleep Quality	The Pittsburg Sleep Quality Index (PSQI; Carpenter & Andrykowski, 1998)	X	X
Symptoms of Relative Energy Deficiency	Relative Energy Deficiency in Sports: RED-S (Edlund Health Q, Edlund, 2020)	X	X

For peer review only

Physical Activity and Sedentary Behavior	Indicator Questions from the Swedish National Board of Health and Welfare	X*	
Bone Density	Dual-Energy X-ray Absorptiometry (DEXA)	X**	

*Physical Activity and Sedentary Behavior will only be performed on the control group.
**DEXA will only be performed on a proportion of climbers.

Eating Disorders Examination-Questionnaire (EDE-Q)

Disordered eating will be measured by the Eating Disorders Examination Questionnaire (EDE-Q 6.0) [23], which is validated and includes 36 questions and records symptoms during the 28 days prior to filling in the questionnaire. EDE-Q consists of four subscales (Restriction Scale, Eating Concern, Figure Concern, and Weight Concern) as well as a score indicating global level of eating disorders symptoms. A cut-off-score of $\geq$ 2.5 will be used as indicative of DE and $\geq$ 4.0 for EDs.

The Oslo Sports Trauma Research Center Overuse Injury Questionnaire (OSTRC-O)

The Oslo Sports Trauma Research Center Overuse Injury Questionnaire (OSTRC-O) [24] is used to gauge the effects of pain and injuries on participation in sport. The original questionnaire measures injuries in the knee, shoulder, foot/lower leg, and lower back. Since climbing is a sport relying heavily on chest, neck, finger, hand, and arm strength, additional items targeting these body parts will be added to the instrument.

Perfectionism

Perfectionism is measured by the Frost Multidimensional Perfectionism Scale (FMPS) [25]. It consists of 35 questions which are measured on a scale from 1-5 depicting the applicability of each statement to the participant. The questions are divided to give scores in six subcategories: Concern over Mistake, Personal Standards, Parental Expectations, Parental Criticism, Doubts about Action, and Organization. A cutoff of >29 points is used in clinical trials [26].

Compulsive Exercise Test (CET)

Compulsive exercise is measured by the Compulsive Exercise Test (CET) [27] which is composed of 24 items with five subscales: avoidance and rule driven behavior, weight control exercise, mood improvement, lack of exercise enjoyment, and exercise rigidity. For CLIMB, only the subscales avoidance and rule-driven behavior and weight control exercise will be included in the analysis since they are deemed the most relevant in athletes.

The Body Shape Questionnaire short version (BSQ-8C)

The Body Shape Questionnaire short version (BSQ-8C) [28] is used to measure body dissatisfaction among participants. This short 8-question version is derived from the original 34-item BSQ, and it is shown to be reliable and valid [29]. We use the Swedish BSQ-8C which has been found to exhibit high internal consistency (α=0.94) as well as excellent test-retest properties [30].

Depression Anxiety and Stress Scale (DASS-21)

Mental health problems are measured by the Depression Anxiety and Stress Scale (DASS-21) [31]. The 21 items consider the week prior to answering the survey and observe symptoms related to depression, anxiety, and stress. The cut-off scores group participants into groups

For peer review only - http://bmjopen.bmj.com/site/about/guidelines.xhtml

showing Normal, Mild, Moderate, Severe, or Extremely Severe symptoms. The exact cut-off values differ in each of the three categories. It is the most widely used version of the survey and boasts test-retest and an internal consistency of α=0.81-0.96 [31, 32].

Pittsburgh Sleep Quality Index (PSQI)
Sleep quality is measured by the Pittsburgh Sleep Quality Index (PSQI) [33] which includes 19 items and evaluates seven subcategories: Subjective sleep quality, sleep latency, sleep duration, habitual sleep efficiency, sleep disturbances, use of sleeping medications, and daytime dysfunction. A Global Score is comprised of these categories on a scale of 0-21, where "0" indicates no sleep difficulty and "21" indicates severe difficulties in all areas.

Symptoms of RED-S will be measured by Edlund Health Q, a 15-item Swedish survey focusing on menstrual cycles, injury, and eating habits.

Physical activity and Sedentary Behavior are measured by two indicator questions established by the Swedish National Board of Health and Welfare (Socialstyrelsen) [34]. The first item is regarding the amount of high intensity activity measured in time per week, such as cardiovascular training. The second item asks the amount of low intensity activity measured in time per week, such as walking or gardening. These two questions are only answered by the control group to establish eligibility among the participants.

DEXA will be used for measuring bone mineral density [35]. This noninvasive imaging technology uses a small amount of ionizing radiation to quantify the amount of bone, fat, and lean tissue, granting the examiners the opportunity to assess the risk of fracture as well as form an overall perception of the bone's objective health and nutritional status.

The web-based questionnaire first introduces the purpose, aims, and ethics of CLIMB, emphasizing the anonymity of the participant. If the participant is <15 years of age, consent from both guardians is required. The questionnaire will be answered by all participants at baseline (climbers and controls), but the follow up questionnaires will only be completed by the climbing group. This is to facilitate future data collection and analysis, as the control group data will only be analyzed at baseline as part of a cross-sectional study. The follow up measurements are therefore only required from the climbers in order to provide longitudinal patterns. DEXA will only be performed on climbers at baseline.

A slightly shorter version of the survey will be used for the follow-up measures at 6, 12, and 24 months (Table 1).

Statistical methods

Based on previous research by one of the authors (UT), a power calculation was estimated on standard deviation of 1.0, significance level set to 0.05, and statistical power to 0.80. The study needs 38 participants in each group to detect estimated group differences. We will therefore include at least 40 participants in each group. It is expected, however, that more than 40 athletes will participate.

Main exposure variables

For peer review only

Disordered eating behavior, body image, overuse injuries/injuries, indicators of low energy availability, mental health problems, compulsive training, perfectionism, sleep quality, sedentary behavior, and bone density.

Outcome

The outcome eating disorder, ED, will be measured with the EDE-Q. The EDE-Q consist of four subscales, restrained eating, eating concern, shape concern and weight concern addressing core dimensions of eating disorders. A score of $\geq$ 4 is indicative of an eating disorder, and for the present study a score of $\geq$ 2.5 will be used as an indication of sub-clinical eating disturbances. Thus, higher scores are indicative of more severe symptoms of eating disorder pathology.

Data analysis plan

Exploratory analyses

Multinominal logistic regression analyses will be used to determine the associations between each of the exposures and trajectories of outcomes. The associations as ORs and 95% CIs will be reported. Bivariate models to measure the crude associations between the exposures and trajectories will be built.

The differences between the climbers and controls regarding the above-mentioned outcome measure *eating disorder*, ED, will be analyzed using multivariate analysis of variance MANOVA. Both within- and between groups analysis will be conducted. Further analyses among climbers with high ED vs low ED will be compared in relation to bone density, injuries, mental health, and symptoms of RED-S. The multivariate analysis of variance MANOVA will be used for that purpose. A one-way repeated measures ANOVA will be used to identify changes over the two-year span in the two groups of climbers with high ED vs. low ED. In cases where overall significant changes are detected, post-hoc analysis will be applied to specify where the between group differences exist.

Structural equation modelling (SEM) will be used at a later stage to investigate if the variables body image, injuries, mental illness, sports environment, bone density (from baseline), and eating disorders can predict injuries.

Ethics and dissemination

All participants will provide informed consent to participate in CLIMB after they had been informed about the purpose and procedure of the study and that it has been approved by the Swedish Ethical Review Authority (reference number: 2021-05557-01).

The dissemination plan is to present our results to the climbing and sport communities, in peer-reviewed scientific journals, at congresses, and to stakeholders with influence in the development of climbing environments.

Trial status

The manuscript reports the protocol (ClinicalTrials.gov NCT05587270) for an ongoing trial for which participants is currently ongoing. Data collection has been initiated and baseline analyses are planned to commence during 2023.

Data Availability Statement

For peer review only - http://bmjopen.bmj.com/site/about/guidelines.xhtml

Data sharing is not applicable to this article as no new data were created or analyzed in this study.

DISCUSSION

Climbing is a weight sensitive sport where athletes generally benefit from a lean body shape and low weight [5]- a risk factor for eating disturbances. Therefore, climbing athletes can be expected to be at high-risk to develop these conditions. There are very few studies regarding eating disturbances among climbers. Due to the overrepresentation of eating disturbances reported in other lean sports and the poor treatment prognosis, early detection and prevention of eating disturbances are essential, especially considering the growing popularity of the sport.

One potential limitation is the use of self-report questionnaires which yield subjective data. Although we will encourage all participants to fill out all surveys, there is the risk of attrition, which we will pay special attention to, using communication strategies at baseline and throughout the follow-up surveys.

There are several major strengths of CLIMB. The first is the use of longitudinal data within several domains related to disturbed eating and mental health issues by using validated questionnaires. The few studies published at this time, on this topic, do not consider multiple mental health parameters that CLIMB intends to address, such as perfectionism, sleep habits, body dissatisfaction, and compulsive exercise. Furthermore, existing studies are cross-sectional and do not follow the participants over a span of several years with incremental measurements, making CLIMB the first of its kind. A control group will be included, which is vital to understanding the results in a societal context.

The use of DEXA is a cornerstone of quality in CLIMB as it complements the participant's self-report data with objective measurements on the effects that Relative Energy Deficiency is associated with. Since osteoporosis has been shown to be a relatively common complication of Anorexia Nervosa [36], and that it is a disease in which pain doesn't manifest until later stages, bringing attention to low bone mineral density among climbers encourages athletes and their support systems to think about the physical, not just mental, potential effects of disturbed eating. DEXA becomes a bridge which leads those involved in the sport to grasp the tangible physical manifestations of mental health issues, establishing a point of action and initiative to prevent and treat afflicted athletes.

CLIMB will be representative of the advanced climbing community at large in Sweden as it includes a wide age span of both genders who climb at sub-elite and elite levels around the country, markedly decreasing the risk of selection bias.

The climbing-concerned scientific community is at the beginning of forming a paradigm around this subject. Combining participant's self-report data with DEXA results has the potential to provide a solid foundation for the continuation of research on this topic. The many facets of this study make CLIMB invaluable in gaining a holistic view on each participant as well as on group levels, which ultimately contributes to developing and implementing evidence-based interventions around the globe.

For peer review only

References:

1. Papageorgiou, M., et al., *Reduced energy availability: implications for bone health in physically active populations.* Eur J Nutr, 2018. **57**(3): p. 847-859.
2. Joubert, L., et al., *Prevalence of amenorrhea in elite female competitive climbers.* Front Sports Act Living, 2022. **4**: p. 895588.
3. Herpertz-Dahlmann, B., *Adolescent eating disorders: update on definitions, symptomatology, epidemiology, and comorbidity.* Child Adolesc Psychiatr Clin N Am, 2015. **24**(1): p. 177-96.
4. Holtkamp, K., J. Hebebrand, and B. Herpertz-Dahlmann, *The contribution of anxiety and food restriction on physical activity levels in acute anorexia nervosa.* Int J Eat Disord, 2004. **36**(2): p. 163-71.
5. Hulley, A.J. and A.J. Hill, *Eating disorders and health in elite women distance runners.* Int J Eat Disord, 2001. **30**(3): p. 312-7.
6. Joy, E., et al., *2014 female athlete triad coalition consensus statement on treatment and return to play of the female athlete triad.* Curr Sports Med Rep, 2014. **13**(4): p. 219-32.
7. Byrne, S. and N. McLean, *Elite athletes: effects of the pressure to be thin.* J Sci Med Sport, 2002. **5**(2): p. 80-94.
8. Werner, A., et al., *Weight-control behaviour and weight-concerns in young elite athletes - a systematic review.* J Eat Disord, 2013. **1**: p. 18.
9. Ravaldi, C., et al., *Eating disorders and body image disturbances among ballet dancers, gymnasium users and body builders.* Psychopathology, 2003. **36**(5): p. 247-54.
10. Mountjoy, M., et al., *The IOC consensus statement: beyond the Female Athlete Triad--Relative Energy Deficiency in Sport (RED-S).* Br J Sports Med, 2014. **48**(7): p. 491-7.
11. Smink, F.R., D. van Hoeken, and H.W. Hoek, *Epidemiology of eating disorders: incidence, prevalence and mortality rates.* Curr Psychiatry Rep, 2012. **14**(4): p. 406-14.
12. Lutter, C., et al., *Sport climbing: medical considerations for this new Olympic discipline.* Br J Sports Med, 2017. **51**(1): p. 2-3.
13. Papageorgiou, M., et al., *Effects of reduced energy availability on bone metabolism in women and men.* Bone, 2017. **105**: p. 191-199.
14. Torstveit, M.K., J.H. Rosenvinge, and J. Sundgot-Borgen, *Prevalence of eating disorders and the predictive power of risk models in female elite athletes: a controlled study.* Scand J Med Sci Sports, 2008. **18**(1): p. 108-18.
15. Stice, E. and H.E. Shaw, *Role of body dissatisfaction in the onset and maintenance of eating pathology: a synthesis of research findings.* J Psychosom Res, 2002. **53**(5): p. 985-93.
16. Sundgot-Borgen, J., *Risk and trigger factors for the development of eating disorders in female elite athletes.* Med Sci Sports Exerc, 1994. **26**(4): p. 414-9.
17. Sundgot-Borgen, J. and M.K. Torstveit, *Aspects of disordered eating continuum in elite high-intensity sports.* Scand J Med Sci Sports, 2010. **20 Suppl 2**: p. 112-21.
18. Sundgot-Borgen, J. and M.K. Torstveit, *Prevalence of eating disorders in elite athletes is higher than in the general population.* Clin J Sport Med, 2004. **14**(1): p. 25-32.
19. Hargreaves, D.A. and M. Tiggemann, *Idealized media images and adolescent body image: "comparing" boys and girls.* Body Image, 2004. **1**(4): p. 351-61.

For peer review only

20. Joy, E., A. Kussman, and A. Nattiv, *2016 update on eating disorders in athletes: A comprehensive narrative review with a focus on clinical assessment and management.* Br J Sports Med, 2016. **50**(3): p. 154-62.
21. Draper, N.e.a., *Comparative grading scales, statistical analyses, climber descriptors and ability grouping: International Rock Climbing Research Association position statement*. 2016, Taylor and Francis: Sports Technology. p. 88-94.
22. Joubert, L.M., G.B. Gonzalez, and A.J. Larson, *Prevalence of Disordered Eating Among International Sport Lead Rock Climbers.* Front Sports Act Living, 2020. **2**: p. 86.
23. Fairburn, C.G. and S.J. Beglin, *Assessment of eating disorders: interview or self-report questionnaire?* Int J Eat Disord, 1994. **16**(4): p. 363-70.
24. Clarsen, B., G. Myklebust, and R. Bahr, *Development and validation of a new method for the registration of overuse injuries in sports injury epidemiology: the Oslo Sports Trauma Research Centre (OSTRC) overuse injury questionnaire.* Br J Sports Med, 2013. **47**(8): p. 495-502.
25. Frost, R., et al., *The dimensions of perfectionism.* 1990: Cognit Ther Res. p. 49-68.
26. Suddarth, B.H. and R.B. & Slaney, *An investigation of the dimensions of perfectionism in college students*. 2001: *Measurement and Evaluation in Counseling and Development*. p. 157–165.
27. Taranis, L., S. Touyz, and C. Meyer, *Disordered eating and exercise: development and preliminary validation of the compulsive exercise test (CET).* Eur Eat Disord Rev, 2011. **19**(3): p. 256-68.
28. Welch, E., M. Lagerström, and A. Ghaderi, *Body shape questionnaire: psychometric properties of the short version (BSQ-8C) and norms from the general Swedish population.* Body Image, 2012. **9**(4): p. 547-50.
29. Evans, C. and B. Dolan, *Body Shape Questionnaire: derivation of shortened "alternate forms".* Int J Eat Disord, 1993. **13**(3): p. 315-21.
30. Edlund, K., et al., *Sustainable UNiversity Life (SUN) study: protocol for a prospective cohort study of modifiable risk and prognostic factors for mental health problems and musculoskeletal pain among university students.* BMJ Open, 2022. **12**(4): p. e056489.
31. Lovibond, P.F. and S.H. Lovibond, *The structure of negative emotional states: comparison of the Depression Anxiety Stress Scales (DASS) with the Beck Depression and Anxiety Inventories.* Behav Res Ther, 1995. **33**(3): p. 335-43.
32. Alfonsson, S., E. Wallin, and P. Maathz, *Factor structure and validity of the Depression, Anxiety and Stress Scale-21 in Swedish translation.* J Psychiatr Ment Health Nurs, 2017. **24**(2-3): p. 154-162.
33. Carpenter, J.S. and M.A. Andrykowski, *Psychometric evaluation of the Pittsburgh Sleep Quality Index.* J Psychosom Res, 1998. **45**(1): p. 5-13.
34. Socialstyrelsen, *Nationella riktlinjer för sjukdomsförebyggande metoder: Tobaksbruk, riskbruk av alkohol, otillräcklig fysisk aktivitet och ohälsosamma matvanor. Stöd för styrning och ledning.* 2011: Stockholm. p. Indikatorer (extern bilaga), p. 14-23.
35. Kim, H.S., et al., *Bone mineral density assessment for research purpose using dual energy X-ray absorptiometry.* Osteoporos Sarcopenia, 2018. **4**(3): p. 79-85.
36. Lennkh, C., et al., *[Osteoporosis in anorexia nervosa. New aspects of pathogenesis and therapy].* Nervenarzt, 1999. **70**(9): p. 823-9.

Authors' contributions

For peer review only - http://bmjopen.bmj.com/site/about/guidelines.xhtml

Dr. Sansone is the project director and Dr. Edlund the co-project director, and they have togeter with the senior researchers Dr. Hedelin and Dr. Tranaeus contributed to the planning of the study as well as the conception and design and have had lead positions in writing this study protocol. Ms. Nigicser and Mr. Identeg are Ph.D-students and Mr. Forsberg a research assistant in the project, and they have contributed to the planning, conception and design of the study including planning and executing the digital survey. They have all contributed to the recruitment of the participants and administered the work around setting up the study. Dr. Tranaeus has also performed the DEXA measures. All authors have contributed to the writing process with input, feedback and critically revising this study protocol. The manuscript was approved by all authors.

Funding statement: This research received no specific grant from any funding agency in the public, commercial or not-for-profit sectors'.

Competing interest statement: The authors declare that they have no competing interests

Word count: 3336

For peer review only

STROBE Statement—Checklist of items that should be included in reports of ***cohort studies***

	Item No	Recommendation	Page No
**Title and abstract**	1	(*a*) Indicate the study's design with a commonly used term in the title or the abstract<br>(*b*) Provide in the abstract an informative and balanced summary of what was done and what was found	1-2
**Introduction**			
Background/rationale	2	Explain the scientific background and rationale for the investigation being reported	2-4
Objectives	3	State specific objectives, including any prespecified hypotheses	4
**Methods**			
Study design	4	Present key elements of study design early in the paper	4
Setting	5	Describe the setting, locations, and relevant dates, including periods of recruitment, exposure, follow-up, and data collection	4-5
Participants	6	(*a*) Give the eligibility criteria, and the sources and methods of selection of participants. Describe methods of follow-up<br>(*b*) For matched studies, give matching criteria and number of exposed and unexposed	4-5
Variables	7	Clearly define all outcomes, exposures, predictors, potential confounders, and effect modifiers. Give diagnostic criteria, if applicable	4-8
Data sources/ measurement	8*	For each variable of interest, give sources of data and details of methods of assessment (measurement). Describe comparability of assessment methods if there is more than one group	4-8
Bias	9	Describe any efforts to address potential sources of bias	n/a
Study size	10	Explain how the study size was arrived at	7
Quantitative variables	11	Explain how quantitative variables were handled in the analyses. If applicable, describe which groupings were chosen and why	n/a
Statistical methods	12	(*a*) Describe all statistical methods, including those used to control for confounding<br>(*b*) Describe any methods used to examine subgroups and interactions<br>(*c*) Explain how missing data were addressed<br>(*d*) If applicable, explain how loss to follow-up was addressed<br>(*e*) Describe any sensitivity analyses	8
**Results**			
Participants	13*	(a) Report numbers of individuals at each stage of study—eg numbers potentially eligible, examined for eligibility, confirmed eligible, included in the study, completing follow-up, and analysed<br>(b) Give reasons for non-participation at each stage<br>(c) Consider use of a flow diagram	n/a
Descriptive data	14*	(a) Give characteristics of study participants (eg demographic, clinical, social) and information on exposures and potential confounders<br>(b) Indicate number of participants with missing data for each variable of interest<br>(c) Summarise follow-up time (eg, average and total amount)	n/a
Outcome data	15*	Report numbers of outcome events or summary measures over time	n/a

For peer review only

For peer review only - http://bmjopen.bmj.com/site/about/guidelines.xhtml

Main results	16	(*a*) Give unadjusted estimates and, if applicable, confounder-adjusted estimates and their precision (eg, 95% confidence interval). Make clear which confounders were adjusted for and why they were included<br>(*b*) Report category boundaries when continuous variables were categorized<br>(*c*) If relevant, consider translating estimates of relative risk into absolute risk for a meaningful time period	n/a
Other analyses	17	Report other analyses done—eg analyses of subgroups and interactions, and sensitivity analyses	n/a
**Discussion**			
Key results	18	Summarise key results with reference to study objectives	n/a
Limitations	19	Discuss limitations of the study, taking into account sources of potential bias or imprecision. Discuss both direction and magnitude of any potential bias	
Interpretation	20	Give a cautious overall interpretation of results considering objectives, limitations, multiplicity of analyses, results from similar studies, and other relevant evidence	n/a
Generalisability	21	Discuss the generalisability (external validity) of the study results	n/a
**Other information**			
Funding	22	Give the source of funding and the role of the funders for the present study and, if applicable, for the original study on which the present article is based	12

*Give information separately for exposed and unexposed groups.

Note: An Explanation and Elaboration article discusses each checklist item and gives methodological background and published examples of transparent reporting. The STROBE checklist is best used in conjunction with this article (freely available on the Web sites of PLoS Medicine at http://www.plosmedicine.org/, Annals of Internal Medicine at http://www.annals.org/, and Epidemiology at http://www.epidem.com/). Information on the STROBE Initiative is available at http://www.strobe-statement.org.

For peer review only

BMJ Open

CLIMB: A Two-Year Longitudinal Study of Eating Disturbances, Mental Health Problems and Overuse Injuries in Rock-climbers: Protocol for a study of risk and prognostic factors.

Journal:	*BMJ Open*
Manuscript ID	bmjopen-2023-074631.R1
Article Type:	Protocol
Date Submitted by the Author:	26-Jul-2023
Complete List of Authors:	Edlund, Klara; Sophiahemmet University, Department of Health Promotion Science; Karolinska Institute, Unit of Intervention Research on Worker Health, Institute of Environmental Medicine<br>Nigicser, Isabel; Sahlgrenska Academy, Ortopedi<br>Sansone, Mikael; Sahlgrenska Academy, Ortopedi<br>Identeg, Fredrik; Sahlgrenska Academy<br>Hedelin, Henrik; Sahlgrenska University Hospital,<br>Forsberg, Niklas; N/A<br>Tranaeus, Ulrika; Swedish School of Sport and Health Sciences, Department of FNB; Karolinska Institute, Institute of Environmental Medicine
**Primary Subject Heading**:	Sports and exercise medicine
Secondary Subject Heading:	Mental health
Keywords:	Eating disorders < PSYCHIATRY, MENTAL HEALTH, SPORTS MEDICINE

SCHOLARONE™
Manuscripts

CLIMB:
A Two-Year Longitudinal Study of Eating Disturbances, Mental Health Problems and Overuse Injuries in Rock-climbers: Protocol for a study of risk and prognostic factors.

Klara Edlund[1,2]
Isabel Nigicser[3]
Mikael Sansone[3]
Fredrik Identeg[3]
Henrik Hedelin[3]
Niklas Forsberg,
Ulrika Tranaeus[4]

Correspondence: Klara Edlund, (ORCID: 0000-0002-2614-5174)

Division of Psychology, Department of Clinical Neuroscience, Karolinska Institutet, SE-171 77, Stockholm, Sweden. E-mail: Klara.edlund@ki.se, phone: +46-706947430

Affiliations

1) Division of Psychology, Department of Clinical Neuroscience, Karolinska Institutet, SE-171 77, Stockholm, Sweden.

2) Musculoskeletal and Sports Injury Epidemiology Center, Department of Health Promotion Sciences, Sophiahemmet University, Stockholm, Sweden.

3) Department of Orthopedics, Institute of Clinical Sciences, Sahlgrenska Academy, University of Gothenburg, Gothenburg, Sweden.

4) Department of Physiology, Nutrition, Biomechanics; Sport Performance & Exercise Research & Innovation Center - Stockholm, SPERIC-S; The Swedish School of Sport and Health Sciences, Stockholm Sweden

For peer review only

ABSTRACT

Objective:
The purpose of the study is to examine eating disorders and overuse injuries in rock-climbers. Furthermore, we want to examine perfectionism, compulsive training, body dissatisfaction, mental health, sleeping, bone density, and relative energy.

Method and analysis:
This prospective longitudinal study aims to recruit Swedish competitive rock-rock-climbers (>13 y o a) via the Swedish Climbing Federation (n=40). A non-athlete control group will be recruited via social media (n=equal of the climbing group). Data will be collected using web-based questionnaires with three follow-ups over two years. Inclusion criteria for rock-climbers will be a minimum advanced level according to IRCRA. The non-athlete control group is matched for age and gender. Exclusion criteria are having competed at an elite level in any sport as well as training more often than twice per week. The primary outcome variables are disordered eating and overuse injuries. Secondary variables are body image, indicators of low energy availability, mental health problems, compulsive training, perfectionism, sleep quality, and bone density. Statistical analyses will include multinominal logistic regression, multivariate analysis of variance MANOVA and Structural equation modelling (SEM). We will assess effect measure modification when relevant and conduct sensitivity analyses to assess the impact of lost to follow-up.

Ethics and dissemination:
The Rock-climbers' Longitudinal attitudes towards Injuries, Mental health, and Body image study, CLIMB, was approved by the Swedish ethics authority (2021-05557-01). Results will be disseminated through peer-reviewed research papers, reports, research conferences, student theses and stakeholder communications.

Trial registration number: NCT05587270.

Article Summary
Strength and limitations of this study:

- Longitudinal assessment of disturbed eating, mental health status, overuse injuries, bone health and symptoms indicative of relative energy deficiency in advanced rock-rock-climbers and normal controls.
- The use of valid and reliable instruments and measurement methods and the large number of potential confounders will improve the internal validity of the estimated associations.
- The use of Dual-Energy X-ray Absorptiometry (DEXA) complements the participant's self-report data with objective measurements on bone health.
- Multivariate statistical analysis will be executed to answer the research questions.
- One limitation is the use of self-report questionnaires only rather than the combination of self-report and structured interviews.

INTRODUCTION

For peer review only

Rock-climbing as a sport places high demands on strength, mobility, and endurance of the athlete. In sports, like rock-climbing, weight and leanness may play an important role for achievement, and there may pressure on athletes with respect to their eating behavior and body weight [1]. In a study by Giel and co-workers [2] on elite athletes in 51 olympic sport disciplines, the results indicated higher rates of eating disorders symptoms among athletes in weight dependent sports as well as higher levels of mental health problems. However, there is limited evidence and mixed results in the literature on eating disorder pathology in elite athletes.

Rock climbing has in recent years gained increased popularity as a recreational and competitive sport, as reflected by a rapid increase of practitioners, media coverage, and the recent inclusion of the sport in the Olympics of 2020 and 2024. Research on climbing related Disordered Eating (DE) and Relative Energy Deficiency in Sports (RED-S) is sparse. The recent increase in the numbers of rock-climbers, both competing athletes and recreational rock-climbers, have brought attention to health issues related to climbing. Especially for high performance climbing, relative strength to weight ratio has been shown to be of importance for performance resulting in lean body shapes, commonly with low BMIs, fat percentages and low calory intakes [3].

Disordered eating is characterized by a deep dissatisfaction with one's own body and/or shape, and is associated with life-threatening medical and psychiatric comorbidities [4].The prevalence of clinical and sub-clinical eating disorders (EDs) in elite athletes have been reported to be 13-16% higher than for the general population [5-7]. In sports where the importance of low body weight is considerable, athletes who compete appear to be particularly vulnerable to the development of disturbed eating patterns [8]. This includes sports such as ballet, gymnastics, and figure-skating [9]. Even at the non-professional level, performers of lean sports emphasizing thinness and muscularity such as body-building and ballet exhibit disordered eating behaviors [10]. Suggested sport-specific, risk factors for eating disturbances include frequent weight regulation, dieting and experienced pressure to lose weight [11, 12]. The International Federation of Sport Climbing (IFSC) has recently implemented a lower weight limit of BMI $\geq$ 17.5 for eligibility to compete in international competition since low body weight (BMI < 17.5) has repeatedly been observed among competitors. To the same end, the Austrian Sport Climbing Organization has set a lower age limit for competitors: 18 years for males, and 17 years for females to mitigate the risk of the developing eating disturbances [13].

RED-S is a syndrome referring to impaired physiological functioning caused by relative energy deficiency and includes, but is not limited to, impairments of metabolic rate, menstrual function, bone health, immunity, protein synthesis and cardiovascular health. Low energy availability (LEA) appears to be an important variable, as studies report that LEA impairs reproductive function and bone formation. As well as decreasing bone formation, it has also been seen to induce a higher increase bone resorption in women compared to men [14]. A recent study by Joubert et al [15] examined 114 female rock-climbers competing at the World Cup level and found that 15.8% presented with current amenorrhea. Additionally, among these rock-climbers with amenorrhea, a larger percent revealed having struggled with DE compared to those without menstrual disturbances (13.5% vs 22.5%, respectively). Studies with a more comprehensive examination of the etiology of RED-S, performance, and injuries among rock-climbers has been called for.

For peer review only

Compulsive exercise (CE) consists of maladaptive compensatory behaviors and serves as a strategy for emotion regulation – often closely linked to eating disturbances. [16]. Weight-affecting behaviors in non-clinical groups have demonstrated addictive qualities that aid the understanding of why these behaviors are difficult to change (Forsén Mantilla et al., 2022). Weight-affecting behaviors such as compulsive exercise have a marked negative effect on psychological and physiological health [17] , and emotion dysregulation has been suggested to trigger these behaviors [18]. A previous study of university students indicated a negative association between symptoms of depression and compulsive exercise for weight control, and a positive association for body dissatisfaction and depression [19]. These results are in line with studies suggesting excessive exercise to be associated with different psychological traits where poor emotion regulation and compulsivity are two of them [20]. This may suggest that compulsive exercise serve as a negatively reinforced behavior, serving as short term alleviation of discomfort. Furthermore, exercising for regulation of negative emotion has been consistently identified as contributory factor to the development and maintenance of EDs [21]. This contribution does not lie specifically in the presence of negative emotion, but rather in the compulsive exercise habits that develop as an outlet for these emotions, which in turn is a risk factor for developing disturbed eating. CE is characterized by an inability or unwillingness to cut down or stop the behavior despite adverse health consequences.

Body dissatisfaction is associated with drive for thinness [22, 23], dieting [24], and disordered eating patterns [24, 25]. The exposure to physical ideals has been seen to have a stronger immediate impact on girls than boys [26], and preoccupation with weight, maladaptive methods for weight control, and body dissatisfaction are known precursors of more serious clinical eating disorders, both among athletes and in the general population [27] [11, 12]. However, athletes from leanness-focused sports report higher rates of eating disturbances compared to athletes from non-weight sensitive sports[9].

Empirical research suggests that elite athletes involved in lean- or weight sensitive sports are at elevated risk of developing an eating disorder (e.g.,[28], [1]). In 2020, one of the first papers on eating disorders and climbing ability was published, which assessed 604 rock-climbers (ages 32 ± 9 years). It presented demographic data according to the International Rock-Climbing Research Association's (IRCRA) directives [29]. The results showed that higher level rock-climbers had significantly lower BMI among both genders, and that 8.6% of the rock-climbers reported disordered eating behaviors. Additionally, 4.2% reported having been treated for an eating disorder and there was an increased risk for eating disorders among female elite rock-climbers compared to males [30]. Strand (2022) [31] states in a study of conversations in climbing communities that conversations related to achieving or maintaining low weight and lean body shape for performance enhancement purposes are common. Further, Strand advises clinicians to be attentive to "weight talk" in the climbing community.

Our main hypothesis is that elite and advanced rock-climbers will present more DE symptoms compared with controls, with elite rock-climbers showing more symptoms than advanced rock-climbers. A secondary hypothesis is that rock-climbers who report more eating disturbances will present with higher levels of health problems including decreased bone density, compulsive exercise, depression, body dissatisfaction, menstrual disturbances and more overuse injuries as compared to rock-climbers with less symptoms of eating disturbances, as these secondary variables are correlates of Eds (e.g., [32]). We expect these patterns to be stable over time. The authors will also examine the gender differences in these categories and expect, for example, to find higher percentages of DE among women than

men, following the pattern of the presented literature. At the time of writing, there exists sparse research on the connection between rock-climbing, disordered eating, and energy deficiency. The presented hypotheses are therefore largely grounded on findings in other sports with similar bodily and/or weight requirements, such as ballet, figure skating, and gymnastics. Due to the rapidly increasing popularity of recreational and competitive climbing, longitudinal cohort studies are therefore urgently needed to understand the etiology of health problems related to eating disorders in rock-climbers in particular. Studies that identify risk- and prognostic factors are required to develop health promotion and disease prevention strategies, which ultimately can reduce the burden of disease in rock-climbers worldwide.

METHOD AND ANALYSIS

Study design and participants

CLIMB will be executed as a prospective longitudinal study, using web-based questionnaires at four occasions (baseline, 6 months, one-, two-, three- and five- years follow-up). The follow-up frequency is established considering that rock-climbing as a sport does not have strict seasons and is highly individualistic, necessitating more frequent measurements. The whole eligible population of Swedish rock-climbers on elite- or advanced levels according to the IRCRA will be invited to participate in the study. Information about the study will be distributed by the Swedish Climbing Federation (https://www.klatterforbundet.se) via climbing gyms and national teams. Information about the study will also be promoted on social media channels such as Facebook and Instagram. Participants will be both male and female, 13 years and older.

The inclusion criteria for the climbing group follow the grade classifications of IRCRA [29] . Each climber must climb at a minimum of advanced level. For women, this means having climbed a boulder route rated harder than 6A or a lead route rated 6c within the last year. For men, this means having climbed a boulder route rated harder than 6B+ or a lead route rated 7a+ within the last year.

A non-athlete control group (n=equal of the climbing group) from the general population, matched in age and gender, will be invited to participate in the study. They will be recruited via social media and universities in Gothenburg and Stockholm, and they will be recruited for cross-sectional analysis only and will not be invited to participate in the follow-ups. The control group's eligibility will be established by answering a two-question survey regarding general physical and sedentary activity (detailed in the Instruments section below), as well as being above 13 years of age. Exclusion factors for a control participant would be to have trained and competed in any sport at the elite level prior to answering the questionnaire, as week as training more than twice per week. One cannot be too inactive to participate in the control group.

Patient and Public Involvement

None.

Instruments

For peer review only

The first portion of the questionnaire aims to confirm that participants are eligible for inclusion, with a climbing level of advanced or above as classified by IRCRA. Demographic data will be gathered and reported including factors such as age, time practicing the sport, amount of weekly training hours, climbing level, and gender. Symptoms related to eating disturbances, RED-S, body image, compulsive training, perfectionism, mental health problems, sleep quality, overuse injuries, and menstrual dysfunction will thereafter be measured (see table 1 below).

In addition to the web-based questionnaire, bone health using Dual-Energy X-ray Absorptiometry (DEXA) will be measured for climbing participants at baseline.

Table 1

Variable	Instrument	Baseline	Follow-up (6 months, one-, two-, three- and five years post baseline)
Eating Disorder Symptoms	The Eating Disorders Questionnaire (EDE-Q 6.0; Fairburn & Beglin, 1994)	X	X
Overuse Injuries	The Oslo Sports Trauma Research Center Overuse Injury Questionnaire (OSTRC-O; Clarsen et al., 2013)	X	X
Perfectionism	The Frost Multidimensional Perfectionism Scale (FMPS; Frost, Marten, Lahart & Rosenblate, 1990)	X	X
Compulsive Training	The Compulsive Exercise Test (CET; Taranis, Touyz & Meyer, 2011)	X	X
Body Dissatisfaction	Body Shape Questionnaire, brief version (BSQ-8C; Welch, Lagerström & Ghaderi, 2012)	X	X
Mental Health	Depression Anxiety Stress Scale-21 (DASS-21; Lovibond & Lovibond, 1995)	X	X
Sleep Quality	The Pittsburg Sleep Quality Index (PSQI; Carpenter & Andrykowski, 1998)	X	X
Symptoms of Relative Energy Deficiency	Relative Energy Deficiency in Sports: RED-S (Edlund Health Q, Edlund, 2020)	X	X
Physical Activity and Sedentary Behavior	Indicator Questions from the Swedish National Board of Health and Welfare	X*	
Bone Density	Dual-Energy X-ray Absorptiometry (DEXA)	X**	

*Physical Activity and Sedentary Behavior will only be performed on the control group.
**DEXA will only be performed on a proportion of rock-climbers.

Eating Disorders Examination-Questionnaire (EDE-Q)

Disordered eating will be measured by the Eating Disorders Examination Questionnaire (EDE-Q 6.0) [33], which is validated and includes 36 questions and records symptoms during the 28 days prior to filling in the questionnaire (e.g., "Have you been deliberately trying to limit the amount of food you eat to influence your shape or weight (whether or not you have succeeded)?". EDE-Q consists of four subscales (Restriction Scale, Eating Concern, Figure

For peer review only

Concern, and Weight Concern) as well as a score indicating global level of eating disorders symptoms. A cut-off-score of ≥ 2.5 will be used as indicative of DE and ≥ 4.0 for EDs. EDE-Q has demonstrated adequate test-retest reliability (r=.89) and internal consistency (α=0.85; [34])

The Oslo Sports Trauma Research Center Overuse Injury Questionnaire (OSTRC-O)

The Oslo Sports Trauma Research Center Overuse Injury Questionnaire (OSTRC-O) [35] is used to gauge the effects of pain and injuries on participation in sport. The original questionnaire measures injuries in the knee, shoulder, foot/lower leg, and lower back. Since climbing is a sport relying heavily on chest, neck, finger, hand, and arm strength, additional items targeting these body parts will be added to the instrument. The questionnaire consists of four questions regarding the athlete's participation in training and competition, reduced training, performance and pain the asked period (e.g., "have you had any difficulties participating in normal training and competition due to (physical) problems last week?" The alternatives for responses are ranged from "full participation without problems", "full participation, but with problems", "reduced participation due to problems" to "cannot participate due to problems". The responses are scored from 0 to 25 for each question with 0 represent no problem and 25 maximal level of the problem. The total score for the OSTRC-0 is 100. The questionnaire shows high internal consistency, with a Cronbach's α of 0.91 [35].

Perfectionism

Perfectionism is measured by the Frost Multidimensional Perfectionism Scale (FMPS) [36]. It consists of 35 questions which are measured on a scale from 1-5 depicting the applicability of each statement to the participant. The questions are divided to give scores in six subcategories: Concern over Mistake, Personal Standards, Parental Expectations, Parental Criticism, Doubts about Action, and Organization (e.g., "It is important to me that I be thoroughly competent in what I do") . A cutoff of >29 points is used in clinical trials [37]. Two subscales will be assessed in the present study due to these subscales showing acceptable to good internal consistency (Personal standards=0.74; Concern over mistakes=0.86; [38]).

Compulsive Exercise Test (CET)

Compulsive exercise is measured by the Compulsive Exercise Test (CET) [39] which is composed of 24 items with five subscales: avoidance and rule driven behavior, weight control exercise, mood improvement, lack of exercise enjoyment, and exercise rigidity (e.g., "I exercise to burn calories and lose weight"). For CLIMB, only the subscales avoidance and rule-driven behavior and weight control exercise will be included in the analysis since they are deemed the most relevant in athletes and have been reported to have adequate validity (factor analysis) and internal consistency (Weight control exercise: a=0.82, Avoidance and rule driven behavior: a=0.87;[40]).

The Body Shape Questionnaire short version (BSQ-8C)

The Body Shape Questionnaire short version (BSQ-8C) [41] is used to measure body dissatisfaction among participants (e.g., "Have you felt so bad about your shape that you have cried?"). This short 8-question version is derived from the original 34-item BSQ, and it is shown to be reliable and valid [42]. We use the Swedish BSQ-8C which has been found to exhibit high internal consistency (α=0.94) as well as excellent test-retest properties [43].

Depression Anxiety and Stress Scale (DASS-21)

Mental health problems are measured by the Depression Anxiety and Stress Scale (DASS-21) [44]. The 21 items consider the week prior to answering the survey and observe symptoms

For peer review only

related to depression, anxiety, and stress (e.g., depression: “I felt that I had nothing to look forward to”; anxiety: “I was aware of dryness of my mouth”; stress: “I found it difficult to relax”). The cut-off scores group participants into groups showing Normal, Mild, Moderate, Severe, or Extremely Severe symptoms. The exact cut-off values differ in each of the three categories. It is the most widely used version of the survey and boasts test-retest and an internal consistency of α=0.81-0.96 [44, 45].

Pittsburgh Sleep Quality Index (PSQI)
Sleep quality is measured by the Pittsburgh Sleep Quality Index (PSQI) [46] which includes 19 items and evaluates seven subcategories: Subjective sleep quality, sleep latency, sleep duration, habitual sleep efficiency, sleep disturbances, use of sleeping medications, and daytime dysfunction (e.g., “ During the past month, how often have you had trouble sleeping because you… wake up in the middle of the night or early morning”) . A Global Score is comprised of these categories on a scale of 0-21, where “0” indicates no sleep difficulty and “21” indicates severe difficulties in all areas. The PSQI has shown adequate internal consistency, reliability and construct validity. Cronbach’s alphas have been reported to 0.80 across groups and correlations between global and component scores have been shown to be moderate to high [46].

Symptoms of RED-S will be measured by Edlund Health Q, a 15-item Swedish survey focusing on menstrual cycles, injury, and eating habits.

Physical activity and Sedentary Behavior are measured by two indicator questions established by the Swedish National Board of Health and Welfare (Socialstyrelsen) [47]. The first item is regarding the amount of high intensity activity measured in time per week, such as cardiovascular training. The second item asks the amount of low intensity activity measured in time per week, such as walking or gardening. These two questions are only answered by the control group to establish eligibility among the participants.

DEXA will be used for measuring bone mineral density [48]. This noninvasive imaging technology uses a small amount of ionizing radiation to quantify the amount of bone, fat, and lean tissue, granting the examiners the opportunity to assess the risk of fracture as well as form an overall perception of the bone’s objective health and nutritional status.

The web-based questionnaire first introduces the purpose, aims, and ethics of CLIMB, emphasizing the anonymity of the participant. If the participant is <15 years of age, consent from both guardians is required. The questionnaire will be answered by all participants at baseline (rock-climbers and controls), but the follow up questionnaires will only be completed by the climbing group. This is to facilitate future data collection and analysis, as the control group data will only be analyzed at baseline as part of a cross-sectional study. The follow up measurements are therefore only required from the rock-climbers in order to provide longitudinal patterns. DEXA will only be performed on rock-climbers at baseline.

Statistical methods

Sample size
No previous data on injury incidence based on overuse injuries exist for rock climbers. Based on previous retrospective data from rock climbers [49], using a power of 80%, a significance

For peer review only

level of 5%, and an expected relative risk of 1.5 for the primary outcome, we calculated that approximately 55 participants was needed in each group (rock climbers and control group).

Main exposure variables

Disordered eating behavior, body image, overuse injuries/injuries, indicators of low energy availability, mental health problems, compulsive training, perfectionism, sleep quality, sedentary behavior, and bone density.

Outcome

The outcome eating disorder, ED, will be measured with the EDE-Q. The EDE-Q consist of four subscales, restrained eating, eating concern, shape concern and weight concern addressing core dimensions of eating disorders. A score of ≥ 4 is indicative of an eating disorder, and for the present study a score of ≥ 2.5 will be used as an indication of sub-clinical eating disturbances. Thus, higher scores are indicative of more severe symptoms of eating disorder pathology.

Our *primary outcomes* are eating disorders symptoms measured with Eating Disorders Examination Questionnaire and overuse injuries measures with OSTRC-O. Our *secondary outcomes* are bone density (DEXA), symptoms of RED-S (Edlund Health Q), mental health problems (Depression, Anxiety and Stress Scale), sleep quality (Pittsburgh Sleep Quality Index), perfectionism (FMPS), compulsive exercise (CET), and body dissatisfaction (BSQ-8).

Data analysis plan

Multinominal logistic regression analyses will be used to determine the associations between each of the exposures and trajectories of outcomes. The associations as ORs and 95% CIs will be reported. Bivariate models to measure the crude associations between the exposures and trajectories will be built.

The differences between the rock-climbers and controls regarding the above-mentioned outcome measure *eating disorder*, ED, will be analyzed using multivariate analysis of variance MANOVA. Both within- and between groups analysis will be conducted. Further analyses among rock-climbers with high ED vs low ED will be compared in relation to bone density, injuries, mental health, and symptoms of RED-S. The multivariate analysis of variance MANOVA will be used for that purpose. A one-way repeated measures ANOVA will be used to identify changes over the two-year span in the two groups of rock-climbers with high ED vs. low ED. In cases where overall significant changes are detected, post-hoc analysis will be applied to specify where the between group differences exist.

Structural equation modelling (SEM) will be used at a later stage to investigate if the variables body image, injuries, mental illness, sports environment, bone density (from baseline), and eating disorders can predict injuries.

Ethics and dissemination

All participants will provide informed consent to participate in CLIMB after they had been informed about the purpose and procedure of the study and that it has been approved by the Swedish Ethical Review Authority (reference number: 2021-05557-01).

For peer review only

The dissemination plan is to present our results to the climbing and sport communities, in peer-reviewed scientific journals, at congresses, and to stakeholders with influence in the development of climbing environments.

Trial status

The manuscript reports the protocol (ClinicalTrials.gov NCT05587270) for an ongoing trial for which participants is currently ongoing. Data collection has been initiated and baseline analyses are planned to commence during 2023. There is a financial plan for the study.

Data Availability Statement
Data sharing is not applicable to this article as no new data were created or analyzed in this study.

DISCUSSION

Climbing is a weight sensitive sport where athletes generally benefit from a lean body shape and low weight [6]- a risk factor for eating disturbances. There are very few studies regarding eating disturbances among rock-climbers. A study by Jouber et. al., examining disordered eating among international sport lead rock climbers, displayed that 6.3 % of male and 16.5 % of female climbers had disordered eating [30]. Similarly, a study examining amenorrhea among female competitive climbers, showed that 15.8% of female climbers had ammenorhea . The IFSC recently released a statement to counteract this development of the sport. Due to the overrepresentation of eating disturbances reported in other lean sports and the poor treatment prognosis, early detection and prevention of eating disturbances and energy deficiency is essential, especially considering the growing popularity of the sport.

There are several major strengths of CLIMB. The first is the use of longitudinal data within several domains related to disturbed eating and mental health issues by using validated questionnaires, reducing misclassification of outcomesThe few studies published at this time, on this topic, do not consider multiple mental health parameters that CLIMB intends to address, such as perfectionism, sleep habits, body dissatisfaction, and compulsive exercise.. It is the first study on rock-climbers which also includes a control group, providing vital context on disease prevalence. CLIMB will be representative of the advanced climbing community at large in Sweden as it includes a wide age span of both genders who climb at advanced and elite levels around Sweden, markedly decreasing the risk of selection bias. Additionally, this large sample of climbers contributes to ensuring external validity.The use of DEXA complements the participant's self-report data with objective measurements on the effects that Relative Energy Deficiency is associated with, establishing a point of action and initiative to prevent and treat afflicted athletes.

One limitation of our study is that we are using a non-random convenience sample that may not be representative to advanced rock-rock-climbers in an international perspective. We have aimed to include the whole advanced level rock-climbing community in Sweden aged 15 years and older, which is the rational for how the participants were recruited. According to the Swedish Climbing Association, there were 15409 climbers affiliated to a climbing club in 2022. It is unknown, however, how many of climbers are of an advanced or higher level, and

For peer review only

thus eligible for participating in the study. Another potential limitation is the use of self-report questionnaires only which yield subjective data. Although the authors will encourage all participants to fill out all surveys using communication strategies at baseline and throughout the follow-up surveys, there is an undeniable risk of increased drop-out rates with each passing survey.

The climbing-concerned scientific community is at the beginning of forming a paradigm around this subject.. The many facets of this study make CLIMB invaluable in gaining a holistic view on each participant as well as on group levels, which ultimately contributes to developing and implementing evidence-based interventions around the globe.

Authors' contributions

Dr. Sansone is the project director and Dr. Edlund the co-project director, and they have together with the senior researchers Dr. Hedelin and Dr. Tranaeus contributed to the planning of the study as well as the conception and design. They, together with PhD students Dr. Nigicser and Dr. Identeg have contributed to writing this study protocol. Mr. Forsberg is a research assistant in the project. They have contributed to the planning, conception and design of the study including planning and executing the digital survey. They have all contributed to the recruitment of the participants and administered the work around setting up the study. Dr. Tranaeus has also performed the DEXA measures. All authors have contributed to the writing process with input, feedback and critically revising this study protocol. The manuscript was approved by all authors.

Funding statement: This research received no specific grant from any funding agency in the public, commercial or not-for-profit sectors.

Competing interest statement: The authors declare that they have no competing interests.

Word count: 3884.

References:

1. Sundgot-Borgen, J., et al., *How to minimise the health risks to athletes who compete in weight-sensitive sports review and position statement on behalf of the Ad Hoc Research Working Group on Body Composition, Health and Performance, under the auspices of the IOC Medical Commission.* Br J Sports Med, 2013. **47**(16): p. 1012-22.
2. Giel, K.E., et al., *Eating disorder pathology in elite adolescent athletes.* Int J Eat Disord, 2016. **49**(6): p. 553-62.
3. Papageorgiou, M., et al., *Reduced energy availability: implications for bone health in physically active populations.* Eur J Nutr, 2018. **57**(3): p. 847-859.
4. Herpertz-Dahlmann, B., *Adolescent eating disorders: update on definitions, symptomatology, epidemiology, and comorbidity.* Child Adolesc Psychiatr Clin N Am, 2015. **24**(1): p. 177-96.

For peer review only

5. Holtkamp, K., J. Hebebrand, and B. Herpertz-Dahlmann, *The contribution of anxiety and food restriction on physical activity levels in acute anorexia nervosa.* Int J Eat Disord, 2004. **36**(2): p. 163-71.
6. Hulley, A.J. and A.J. Hill, *Eating disorders and health in elite women distance runners.* Int J Eat Disord, 2001. **30**(3): p. 312-7.
7. Joy, E., et al., *2014 female athlete triad coalition consensus statement on treatment and return to play of the female athlete triad.* Curr Sports Med Rep, 2014. **13**(4): p. 219-32.
8. Byrne, S. and N. McLean, *Elite athletes: effects of the pressure to be thin.* J Sci Med Sport, 2002. **5**(2): p. 80-94.
9. Werner, A., et al., *Weight-control behaviour and weight-concerns in young elite athletes - a systematic review.* J Eat Disord, 2013. **1**: p. 18.
10. Ravaldi, C., et al., *Eating disorders and body image disturbances among ballet dancers, gymnasium users and body builders.* Psychopathology, 2003. **36**(5): p. 247-54.
11. Mountjoy, M., et al., *The IOC consensus statement: beyond the Female Athlete Triad--Relative Energy Deficiency in Sport (RED-S).* Br J Sports Med, 2014. **48**(7): p. 491-7.
12. Smink, F.R., D. van Hoeken, and H.W. Hoek, *Epidemiology of eating disorders: incidence, prevalence and mortality rates.* Curr Psychiatry Rep, 2012. **14**(4): p. 406-14.
13. Lutter, C., et al., *Sport climbing: medical considerations for this new Olympic discipline.* Br J Sports Med, 2017. **51**(1): p. 2-3.
14. Papageorgiou, M., et al., *Effects of reduced energy availability on bone metabolism in women and men.* Bone, 2017. **105**: p. 191-199.
15. Joubert, L., et al., *Prevalence of amenorrhea in elite female competitive climbers.* Front Sports Act Living, 2022. **4**: p. 895588.
16. Forsén Mantilla, E., et al., *Impulsivity and compulsivity as parallel mediators of emotion dysregulation in eating-related addictive-like behaviors, alcohol use, and compulsive exercise.* Brain Behav, 2022. **12**(1): p. e2458.
17. Godier, L.R. and R.J. Park, *A novel measure of compulsive food restriction in anorexia nervosa: validation of the Self-Starvation Scale (SS).* Eat Behav, 2015. **17**: p. 10-3.
18. Bunio, L.K., *The nuances of emotion regulation difficulties and mindfulness in food addiction*, J.A. Battles, Loverich, Tamara M., Editor. 2021, Addiction Research & Theory. p. 11-17.
19. Edlund, K., et al., *Body image and compulsive exercise: are there associations with depression among university students?* Eat Weight Disord, 2022. **27**(7): p. 2397-2405.
20. Dyrbye, L.N., M.R. Thomas, and T.D. Shanafelt, *Systematic review of depression, anxiety, and other indicators of psychological distress among U.S. and Canadian medical students.* Acad Med, 2006. **81**(4): p. 354-73.
21. Torstveit, M.K., J.H. Rosenvinge, and J. Sundgot-Borgen, *Prevalence of eating disorders and the predictive power of risk models in female elite athletes: a controlled study.* Scand J Med Sci Sports, 2008. **18**(1): p. 108-18.

For peer review only

22. Stice, E. and H.E. Shaw, *Role of body dissatisfaction in the onset and maintenance of eating pathology: a synthesis of research findings.* J Psychosom Res, 2002. **53**(5): p. 985-93.
23. Sundgot-Borgen, J., *Risk and trigger factors for the development of eating disorders in female elite athletes.* Med Sci Sports Exerc, 1994. **26**(4): p. 414-9.
24. Sundgot-Borgen, J. and M.K. Torstveit, *Aspects of disordered eating continuum in elite high-intensity sports.* Scand J Med Sci Sports, 2010. **20 Suppl 2**: p. 112-21.
25. Sundgot-Borgen, J. and M.K. Torstveit, *Prevalence of eating disorders in elite athletes is higher than in the general population.* Clin J Sport Med, 2004. **14**(1): p. 25-32.
26. Hargreaves, D.A. and M. Tiggemann, *Idealized media images and adolescent body image: "comparing" boys and girls.* Body Image, 2004. **1**(4): p. 351-61.
27. Joy, E., A. Kussman, and A. Nattiv, *2016 update on eating disorders in athletes: A comprehensive narrative review with a focus on clinical assessment and management.* Br J Sports Med, 2016. **50**(3): p. 154-62.
28. Bar, R.J., S.E. Cassin, and M.M. Dionne, *Eating disorder prevention initiatives for athletes: A review.* Eur J Sport Sci, 2016. **16**(3): p. 325-35.
29. Draper, N.e.a., *Comparative grading scales, statistical analyses, climber descriptors and ability grouping:*
International Rock Climbing Research Association position statement. 2016, Taylor and Francis: Sports Technology. p. 88-94.
30. Joubert, L.M., G.B. Gonzalez, and A.J. Larson, *Prevalence of Disordered Eating Among International Sport Lead Rock Climbers.* Front Sports Act Living, 2020. **2**: p. 86.
31. Strand, M., *Attitudes towards disordered eating in the rock climbing community: a digital ethnography.* J Eat Disord, 2022. **10**(1): p. 96.
32. Mountjoy, M., et al., *International Olympic Committee (IOC) Consensus Statement on Relative Energy Deficiency in Sport (RED-S): 2018 Update.* Int J Sport Nutr Exerc Metab, 2018. **28**(4): p. 316-331.
33. Fairburn, C.G. and S.J. Beglin, *Assessment of eating disorders: interview or self-report questionnaire?* Int J Eat Disord, 1994. **16**(4): p. 363-70.
34. Carter, J.C., D.A. Stewart, and C.G. Fairburn, *Eating disorder examination questionnaire: norms for young adolescent girls.* Behav Res Ther, 2001. **39**(5): p. 625-32.
35. Clarsen, B., G. Myklebust, and R. Bahr, *Development and validation of a new method for the registration of overuse injuries in sports injury epidemiology: the Oslo Sports Trauma Research Centre (OSTRC) overuse injury questionnaire.* Br J Sports Med, 2013. **47**(8): p. 495-502.
36. Frost, R., et al., *The dimensions of perfectionism.* 1990: Cognit Ther Res. p. 49-68.
37. Suddarth, B.H. and R.B. & Slaney, *An investigation of the dimensions of perfectionism in college students*. 2001: *Measurement and Evaluation in Counseling and Development*. p. 157–165.
38. Bußenius, L. and S. Harendza, *The relationship between perfectionism and symptoms of depression in medical school applicants.* BMC Med Educ, 2019. **19**(1): p. 370.

For peer review only

39. Taranis, L., S. Touyz, and C. Meyer, *Disordered eating and exercise: development and preliminary validation of the compulsive exercise test (CET).* Eur Eat Disord Rev, 2011. **19**(3): p. 256-68.

40. Plateau, C., *Use of the compulsive exercise test with athletes: Norms and links with eating psychopathology.*, V. Shanmugam, Duckham RL., Editor. 2014, Journal of Applied Sport Psychology. p. 287-301.

41. Welch, E., M. Lagerström, and A. Ghaderi, *Body shape questionnaire: psychometric properties of the short version (BSQ-8C) and norms from the general Swedish population.* Body Image, 2012. **9**(4): p. 547-50.

42. Evans, C. and B. Dolan, *Body Shape Questionnaire: derivation of shortened "alternate forms".* Int J Eat Disord, 1993. **13**(3): p. 315-21.

43. Edlund, K., et al., *Sustainable UNiversity Life (SUN) study: protocol for a prospective cohort study of modifiable risk and prognostic factors for mental health problems and musculoskeletal pain among university students.* BMJ Open, 2022. **12**(4): p. e056489.

44. Lovibond, P.F. and S.H. Lovibond, *The structure of negative emotional states: comparison of the Depression Anxiety Stress Scales (DASS) with the Beck Depression and Anxiety Inventories.* Behav Res Ther, 1995. **33**(3): p. 335-43.

45. Alfonsson, S., E. Wallin, and P. Maathz, *Factor structure and validity of the Depression, Anxiety and Stress Scale-21 in Swedish translation.* J Psychiatr Ment Health Nurs, 2017. **24**(2-3): p. 154-162.

46. Carpenter, J.S. and M.A. Andrykowski, *Psychometric evaluation of the Pittsburgh Sleep Quality Index.* J Psychosom Res, 1998. **45**(1): p. 5-13.

47. Socialstyrelsen, *Nationella riktlinjer för sjukdomsförebyggande metoder: Tobaksbruk, riskbruk av alkohol, otillräcklig fysisk aktivitet och ohälsosamma matvanor. Stöd för styrning och ledning.* 2011: Stockholm. p. Indikatorer (extern bilaga), p. 14-23.

48. Kim, H.S., et al., *Bone mineral density assessment for research purpose using dual energy X-ray absorptiometry.* Osteoporos Sarcopenia, 2018. **4**(3): p. 79-85.

49. Jones, G., D. Llewellyn, and M.I. Johnson, *Previous injury as a risk factor for reinjury in rock climbing: a secondary analysis of data from a retrospective cross-sectional cohort survey of active rock climbers.* BMJ Open Sport Exerc Med, 2015. **1**(1).

For peer review only

For peer review only - http://bmjopen.bmj.com/site/about/guidelines.xhtml

STROBE Statement—Checklist of items that should be included in reports of ***cohort studies***

	Item No	Recommendation	Page No
**Title and abstract**	1	(*a*) Indicate the study's design with a commonly used term in the title or the abstract<br>(*b*) Provide in the abstract an informative and balanced summary of what was done and what was found	1-2
**Introduction**			
Background/rationale	2	Explain the scientific background and rationale for the investigation being reported	2-4
Objectives	3	State specific objectives, including any prespecified hypotheses	4
**Methods**			
Study design	4	Present key elements of study design early in the paper	4
Setting	5	Describe the setting, locations, and relevant dates, including periods of recruitment, exposure, follow-up, and data collection	4-5
Participants	6	(*a*) Give the eligibility criteria, and the sources and methods of selection of participants. Describe methods of follow-up<br>(*b*) For matched studies, give matching criteria and number of exposed and unexposed	4-5
Variables	7	Clearly define all outcomes, exposures, predictors, potential confounders, and effect modifiers. Give diagnostic criteria, if applicable	4-8
Data sources/ measurement	8*	For each variable of interest, give sources of data and details of methods of assessment (measurement). Describe comparability of assessment methods if there is more than one group	4-8
Bias	9	Describe any efforts to address potential sources of bias	n/a
Study size	10	Explain how the study size was arrived at	7
Quantitative variables	11	Explain how quantitative variables were handled in the analyses. If applicable, describe which groupings were chosen and why	n/a
Statistical methods	12	(*a*) Describe all statistical methods, including those used to control for confounding<br>(*b*) Describe any methods used to examine subgroups and interactions<br>(*c*) Explain how missing data were addressed<br>(*d*) If applicable, explain how loss to follow-up was addressed<br>(*e*) Describe any sensitivity analyses	8
**Results**			
Participants	13*	(a) Report numbers of individuals at each stage of study—eg numbers potentially eligible, examined for eligibility, confirmed eligible, included in the study, completing follow-up, and analysed<br>(b) Give reasons for non-participation at each stage<br>(c) Consider use of a flow diagram	n/a
Descriptive data	14*	(a) Give characteristics of study participants (eg demographic, clinical, social) and information on exposures and potential confounders<br>(b) Indicate number of participants with missing data for each variable of interest<br>(c) Summarise follow-up time (eg, average and total amount)	n/a
Outcome data	15*	Report numbers of outcome events or summary measures over time	n/a

For peer review only

Main results	16	(*a*) Give unadjusted estimates and, if applicable, confounder-adjusted estimates and their precision (eg, 95% confidence interval). Make clear which confounders were adjusted for and why they were included<br>(*b*) Report category boundaries when continuous variables were categorized<br>(*c*) If relevant, consider translating estimates of relative risk into absolute risk for a meaningful time period	n/a
Other analyses	17	Report other analyses done—eg analyses of subgroups and interactions, and sensitivity analyses	n/a
**Discussion**			
Key results	18	Summarise key results with reference to study objectives	n/a
Limitations	19	Discuss limitations of the study, taking into account sources of potential bias or imprecision. Discuss both direction and magnitude of any potential bias	
Interpretation	20	Give a cautious overall interpretation of results considering objectives, limitations, multiplicity of analyses, results from similar studies, and other relevant evidence	n/a
Generalisability	21	Discuss the generalisability (external validity) of the study results	n/a
**Other information**			
Funding	22	Give the source of funding and the role of the funders for the present study and, if applicable, for the original study on which the present article is based	12

*Give information separately for exposed and unexposed groups.

Note: An Explanation and Elaboration article discusses each checklist item and gives methodological background and published examples of transparent reporting. The STROBE checklist is best used in conjunction with this article (freely available on the Web sites of PLoS Medicine at http://www.plosmedicine.org/, Annals of Internal Medicine at http://www.annals.org/, and Epidemiology at http://www.epidem.com/). Information on the STROBE Initiative is available at http://www.strobe-statement.org.

For peer review only

BMJ Open

Protocol for a Two-Year Longitudinal Study of Eating Disturbances, Mental Health Problems and Overuse Injuries in Rock-Climbers (CLIMB)

Journal:	*BMJ Open*
Manuscript ID	bmjopen-2023-074631.R2
Article Type:	Protocol
Date Submitted by the Author:	21-Aug-2023
Complete List of Authors:	Edlund, Klara; Sophiahemmet University, Department of Health Promotion Science; Karolinska Institute, Unit of Intervention Research on Worker Health, Institute of Environmental Medicine<br>Nigicser, Isabel; Sahlgrenska Academy, Ortopedi<br>Sansone, Mikael; Sahlgrenska Academy, Ortopedi<br>Identeg, Fredrik; Sahlgrenska Academy<br>Hedelin, Henrik; Sahlgrenska University Hospital,<br>Forsberg, Niklas; N/A<br>Tranaeus, Ulrika; Swedish School of Sport and Health Sciences, Department of FNB; Karolinska Institute, Institute of Environmental Medicine
**Primary Subject Heading**:	Sports and exercise medicine
Secondary Subject Heading:	Mental health
Keywords:	Eating disorders < PSYCHIATRY, MENTAL HEALTH, SPORTS MEDICINE

SCHOLARONE™
Manuscripts

Protocol for a Two-Year Longitudinal Study of Eating Disturbances, Mental Health Problems and Overuse Injuries in Rock-Climbers (CLIMB)

For peer review only

Klara Edlund[1,2]
Isabel Nigicser[3]
Mikael Sansone[3]
Fredrik Identeg[3]
Henrik Hedelin[3]
Niklas Forsberg,
Ulrika Tranaeus[4]

Correspondence: Klara Edlund, (ORCID: 0000-0002-2614-5174)

Division of Psychology, Department of Clinical Neuroscience, Karolinska Institutet, SE-171 77, Stockholm, Sweden. E-mail: Klara.edlund@ki.se, phone: +46-706947430

Affiliations

1) Division of Psychology, Department of Clinical Neuroscience, Karolinska Institutet, SE-171 77, Stockholm, Sweden.

2) Musculoskeletal and Sports Injury Epidemiology Center, Department of Health Promotion Sciences, Sophiahemmet University, Stockholm, Sweden.

3) Department of Orthopedics, Institute of Clinical Sciences, Sahlgrenska Academy, University of Gothenburg, Gothenburg, Sweden.

4) Department of Physiology, Nutrition, Biomechanics; Sport Performance & Exercise Research & Innovation Center - Stockholm, SPERIC-S; The Swedish School of Sport and Health Sciences, Stockholm Sweden

ABSTRACT

Introduction:

Rock-climbing is a rapidly growing sport in which performance may be affected by participant's weight and leanness, and there may pressure on athletes with respect to their eating behavior and body weight. However, there is sparse research performed on climbers, constituting a knowledge gap which the present study aims to fill. The primary outcomes of the study are to examine disordered eating and overuse injuries in rock-climbers. Secondary variables are body image, indicators of relative energy deficiency, mental health problems, compulsive training, perfectionism, sleep quality, and bone density.

Method and analysis:

This prospective longitudinal study aims to recruit Swedish competitive rock-climbers (>13 y o a) via the Swedish Climbing Federation. A non-athlete control group will be recruited via social media (n=equal of the climbing group). Data will be collected using streamlined validated web-based questionnaires with three follow-ups over two years. Inclusion criteria for rock-climbers will be a minimum advanced level according to IRCRA. The non-athlete control group is matched for age and gender. Exclusion criteria are having competed at an elite level in any sport as well as training more often than twice per week. Statistical analyses will include multinominal logistic regression, multivariate analysis of variance MANOVA and Structural equation modelling (SEM). We will assess effect measure modification when relevant and conduct sensitivity analyses to assess the impact of lost to follow-up.

Ethics and dissemination:

The Rock-Climbers' Longitudinal attitudes towards Injuries, Mental health, and Body image study, CLIMB, was approved by the Swedish ethics authority (2021-05557-01). Results will be disseminated through peer-reviewed research papers, reports, research conferences, student theses and stakeholder communications.

Trial registration number: NCT05587270.

Article Summary

Strength and limitations of this study:

- Longitudinal assessment of disturbed eating, mental health status, overuse injuries, bone health and symptoms indicative of relative energy deficiency in advanced rock-rock-climbers and normal controls.
- The use of valid and reliable instruments and measurement methods and the large number of potential confounders will improve the internal validity of the estimated associations.
- The use of Dual-Energy X-ray Absorptiometry (DEXA) complements the participant's self-report data with objective measurements on bone health.
- Multivariate statistical analysis will be executed to answer the research questions.
- One limitation is the use of self-report questionnaires only rather than the combination of self-report and structured interviews.

For peer review only

INTRODUCTION

Rock-climbing as a sport places high demands on strength, mobility, and endurance of the athlete. In sports, like rock-climbing, weight and leanness may play an important role for achievement, and there may pressure on athletes with respect to their eating behavior and body weight [1]. In a study by Giel and co-workers [2] on elite athletes in 51 Olympic sport disciplines, the results indicated higher rates of eating disorders symptoms among athletes in weight dependent sports as well as higher levels of mental health problems. However, there is limited evidence and mixed results in the literature on eating disorder pathology in elite athletes.

Rock climbing has in recent years gained increased popularity as a recreational and competitive sport, as reflected by a rapid increase of practitioners, media coverage, and the recent inclusion of the sport in the Olympics of 2020 and 2024. Research on climbing related Disordered Eating (DE) and Relative Energy Deficiency in Sports (RED-S) is sparse. The recent increase in the numbers of rock-climbers, both competing athletes and recreational rock-climbers, have brought attention to health issues related to climbing. Especially for high performance climbing, relative strength to weight ratio has been shown to be of importance for performance resulting in lean body shapes, commonly with low BMIs, fat percentages and low calory intakes [3].

Disordered eating is characterized by a deep dissatisfaction with one's own body and/or shape, and is associated with life-threatening medical and psychiatric comorbidities [4].The prevalence of clinical and sub-clinical eating disorders (EDs) in elite athletes have been reported to be 13-16% higher than for the general population [5-7]. In sports where the importance of low body weight is considerable, athletes who compete appear to be particularly vulnerable to the development of disturbed eating patterns [8]. This includes sports such as ballet, gymnastics, and figure-skating [9]. Even at the non-professional level, performers of lean sports emphasizing thinness and muscularity such as body-building and ballet exhibit disordered eating behaviors [10]. Suggested sport-specific, risk factors for eating disturbances include frequent weight regulation, dieting and experienced pressure to lose weight [11, 12]. The International Federation of Sport Climbing (IFSC) has recently implemented a lower weight limit of BMI $\geq$ 17.5 for eligibility to compete in international competition since low body weight (BMI < 17.5) has repeatedly been observed among competitors. To the same end, the Austrian Sport Climbing Organization has set a lower age limit for competitors: 18 years for males, and 17 years for females to mitigate the risk of the developing eating disturbances [13].

RED-S is a syndrome referring to impaired physiological functioning caused by relative energy deficiency and includes, but is not limited to, impairments of metabolic rate, menstrual function, bone health, immunity, protein synthesis and cardiovascular health. Low energy availability (LEA) appears to be an important variable, as studies report that LEA impairs reproductive function and bone formation. As well as decreasing bone formation, it has also been seen to induce a higher increase bone resorption in women compared to men [14]. A recent study by Joubert et al [15] examined 114 female rock-climbers competing at the World Cup level and found that 15.8% presented with current amenorrhea. Additionally, among these rock-climbers with amenorrhea, a larger percent revealed having struggled with DE compared to those without menstrual disturbances (13.5% vs 22.5%, respectively). Studies with a more comprehensive examination of the etiology of RED-S, performance, and injuries among rock-climbers has been called for.

For peer review only

Compulsive exercise (CE) consists of maladaptive compensatory behaviors and serves as a strategy for emotion regulation – often closely linked to eating disturbances. [16]. Weight-affecting behaviors in non-clinical groups have demonstrated addictive qualities that aid the understanding of why these behaviors are difficult to change (Forsén Mantilla et al., 2022). Weight-affecting behaviors such as compulsive exercise have a marked negative effect on psychological and physiological health [17] , and emotion dysregulation has been suggested to trigger these behaviors [18]. A previous study of university students indicated a negative association between symptoms of depression and compulsive exercise for weight control, and a positive association for body dissatisfaction and depression [19]. These results are in line with studies suggesting excessive exercise to be associated with different psychological traits where poor emotion regulation and compulsivity are two of them [20]. This may suggest that compulsive exercise serve as a negatively reinforced behavior, serving as short term alleviation of discomfort. Furthermore, exercising for regulation of negative emotion has been consistently identified as contributory factor to the development and maintenance of EDs [21]. This contribution does not lie specifically in the presence of negative emotion, but rather in the compulsive exercise habits that develop as an outlet for these emotions, which in turn is a risk factor for developing disturbed eating. CE is characterized by an inability or unwillingness to cut down or stop the behavior despite adverse health consequences.

Body dissatisfaction is associated with drive for thinness [22, 23], dieting [24], and disordered eating patterns [24, 25]. The exposure to physical ideals has been seen to have a stronger immediate impact on girls than boys [26], and preoccupation with weight, maladaptive methods for weight control, and body dissatisfaction are known precursors of more serious clinical eating disorders, both among athletes and in the general population [27] [11, 12]. However, athletes from leanness-focused sports report higher rates of eating disturbances compared to athletes from non-weight sensitive sports[9].

Empirical research suggests that elite athletes involved in lean- or weight sensitive sports are at elevated risk of developing an eating disorder (e.g.,[28], [1]). In 2020, one of the first papers on eating disorders and climbing ability was published, which assessed 604 rock-climbers (ages 32 ± 9 years). It presented demographic data according to the International Rock-Climbing Research Association's (IRCRA) directives [29]. The results showed that higher level rock-climbers had significantly lower BMI among both genders, and that 8.6% of the rock-climbers reported disordered eating behaviors. Additionally, 4.2% reported having been treated for an eating disorder and there was an increased risk for eating disorders among female elite rock-climbers compared to males [30]. Strand (2022) [31] states in a study of conversations in climbing communities that conversations related to achieving or maintaining low weight and lean body shape for performance enhancement purposes are common. Further, Strand advises clinicians to be attentive to "weight talk" in the climbing community.

Our main hypothesis is that elite and advanced rock-climbers will present more DE symptoms compared with controls, with elite rock-climbers showing more symptoms than advanced rock-climbers. A secondary hypothesis is that rock-climbers who report more eating disturbances will present with higher levels of health problems including decreased bone density, compulsive exercise, depression, body dissatisfaction, menstrual disturbances and more overuse injuries as compared to rock-climbers with less symptoms of eating disturbances, as these secondary variables are correlates of Eds (e.g., [32]). We expect these patterns to be stable over time. The authors will also examine the gender differences in these categories and expect, for example, to find higher percentages of DE among women than

For peer review only

men, following the pattern of the presented literature. At the time of writing, there exists sparse research on the connection between rock-climbing, disordered eating, and energy deficiency. The presented hypotheses are therefore largely grounded on findings in other sports with similar bodily and/or weight requirements, such as ballet, figure skating, and gymnastics. Due to the rapidly increasing popularity of recreational and competitive climbing, longitudinal cohort studies are therefore urgently needed to understand the etiology of health problems related to eating disorders in rock-climbers in particular. Studies that identify risk- and prognostic factors are required to develop health promotion and disease prevention strategies, which ultimately can reduce the burden of disease in rock-climbers worldwide.

METHOD AND ANALYSIS

Study design and participants

CLIMB will be executed as a prospective longitudinal study, using web-based questionnaires at four occasions (baseline, 6 months, one-, two-, three- and five- years follow-up). The follow-up frequency is established considering that rock-climbing as a sport does not have strict seasons and is highly individualistic, necessitating more frequent measurements. The whole eligible population of Swedish rock-climbers on elite- or advanced levels according to the IRCRA will be invited to participate in the study. Information about the study will be distributed by the Swedish Climbing Federation (https://www.klatterforbundet.se) via climbing gyms and national teams. Information about the study will also be promoted on social media channels such as Facebook and Instagram. Participants will be both male and female, 13 years and older.

The inclusion criteria for the climbing group follow the grade classifications of IRCRA [29] . Each climber must climb at a minimum of advanced level. For women, this means having climbed a boulder route rated harder than 6A or a lead route rated 6c within the last year. For men, this means having climbed a boulder route rated harder than 6B+ or a lead route rated 7a+ within the last year.

A non-athlete control group (n=equal of the climbing group) from the general population, matched in age and gender, will be invited to participate in the study. They will be recruited via social media and universities in Gothenburg and Stockholm, and they will be recruited for cross-sectional analysis only and will not be invited to participate in the follow-ups. The control group's eligibility will be established by answering a two-question survey regarding general physical and sedentary activity (detailed in the Instruments section below), as well as being above 13 years of age. Exclusion factors for a control participant would be to have trained and competed in any sport at the elite level prior to answering the questionnaire, as week as training more than twice per week. One cannot be too inactive to participate in the control group.

Patient and Public Involvement

None.

Instruments

For peer review only

The first portion of the questionnaire aims to confirm that participants are eligible for inclusion, with a climbing level of advanced or above as classified by IRCRA. Demographic data will be gathered and reported including factors such as age, time practicing the sport, amount of weekly training hours, climbing level, and gender. Symptoms related to eating disturbances, RED-S, body image, compulsive training, perfectionism, mental health problems, sleep quality, overuse injuries, and menstrual dysfunction will thereafter be measured (see table 1 below).

In addition to the web-based questionnaire, bone health using Dual-Energy X-ray Absorptiometry (DEXA) will be measured for climbing participants at baseline.

Table 1

Variable	Instrument	Baseline	Follow-up (6 months, one-, two-, three- and five years post baseline)
Eating Disorder Symptoms	The Eating Disorders Questionnaire (EDE-Q 6.0; Fairburn & Beglin, 1994)	X	X
Overuse Injuries	The Oslo Sports Trauma Research Center Overuse Injury Questionnaire (OSTRC-O; Clarsen et al., 2013)	X	X
Perfectionism	The Frost Multidimensional Perfectionism Scale (FMPS; Frost, Marten, Lahart & Rosenblate, 1990)	X	X
Compulsive Training	The Compulsive Exercise Test (CET; Taranis, Touyz & Meyer, 2011)	X	X
Body Dissatisfaction	Body Shape Questionnaire, brief version (BSQ-8C; Welch, Lagerström & Ghaderi, 2012)	X	X
Mental Health	Depression Anxiety Stress Scale-21 (DASS-21; Lovibond & Lovibond, 1995)	X	X
Sleep Quality	The Pittsburg Sleep Quality Index (PSQI; Carpenter & Andrykowski, 1998)	X	X
Symptoms of Relative Energy Deficiency	Relative Energy Deficiency in Sports: RED-S (Edlund Health Q, Edlund, 2020)	X	X
Physical Activity and Sedentary Behavior	Indicator Questions from the Swedish National Board of Health and Welfare	X*	
Bone Density	Dual-Energy X-ray Absorptiometry (DEXA)	X**	

*Physical Activity and Sedentary Behavior will only be performed on the control group.
**DEXA will only be performed on a proportion of rock-climbers.

Eating Disorders Examination-Questionnaire (EDE-Q)

Disordered eating will be measured by the Eating Disorders Examination Questionnaire (EDE-Q 6.0) [33], which is validated and includes 36 questions and records symptoms during the 28 days prior to filling in the questionnaire (e.g., "Have you been deliberately trying to limit the amount of food you eat to influence your shape or weight (whether or not you have succeeded)?". EDE-Q consists of four subscales (Restriction Scale, Eating Concern, Figure Concern, and Weight Concern) as well as a score indicating global level of eating disorders symptoms. A cut-off-score of $\geq$ 2.5 will be used as indicative of DE and $\geq$ 4.0 for EDs. EDE-

For peer review only

Q has demonstrated adequate test-retest reliability (r=.89) and internal consistency (α=0.85; [34])

The Oslo Sports Trauma Research Center Overuse Injury Questionnaire (OSTRC-O)

The Oslo Sports Trauma Research Center Overuse Injury Questionnaire (OSTRC-O) [35] is used to gauge the effects of pain and injuries on participation in sport. The original questionnaire measures injuries in the knee, shoulder, foot/lower leg, and lower back. Since climbing is a sport relying heavily on chest, neck, finger, hand, and arm strength, additional items targeting these body parts will be added to the instrument. The questionnaire consists of four questions regarding the athlete's participation in training and competition, reduced training, performance and pain the asked period (e.g., "have you had any difficulties participating in normal training and competition due to (physical) problems last week?" The alternatives for responses are ranged from "full participation without problems", "full participation, but with problems", "reduced participation due to problems" to "cannot participate due to problems". The responses are scored from 0 to 25 for each question with 0 represent no problem and 25 maximal level of the problem. The total score for the OSTRC-0 is 100. The questionnaire shows high internal consistency, with a Cronbach's α of 0.91 [35].

Perfectionism

Perfectionism is measured by the Frost Multidimensional Perfectionism Scale (FMPS) [36]. It consists of 35 questions which are measured on a scale from 1-5 depicting the applicability of each statement to the participant. The questions are divided to give scores in six subcategories: Concern over Mistake, Personal Standards, Parental Expectations, Parental Criticism, Doubts about Action, and Organization (e.g., "It is important to me that I be thoroughly competent in what I do") . A cutoff of >29 points is used in clinical trials [37]. Two subscales will be assessed in the present study due to these subscales showing acceptable to good internal consistency (Personal standards=0.74; Concern over mistakes=0.86; [38]).

Compulsive Exercise Test (CET)

Compulsive exercise is measured by the Compulsive Exercise Test (CET) [39] which is composed of 24 items with five subscales: avoidance and rule driven behavior, weight control exercise, mood improvement, lack of exercise enjoyment, and exercise rigidity (e.g., "I exercise to burn calories and lose weight"). For CLIMB, only the subscales avoidance and rule-driven behavior and weight control exercise will be included in the analysis since they are deemed the most relevant in athletes and have been reported to have adequate validity (factor analysis) and internal consistency (Weight control exercise: a=0.82, Avoidance and rule driven behavior: a=0.87;[40]).

The Body Shape Questionnaire short version (BSQ-8C)

The Body Shape Questionnaire short version (BSQ-8C) [41] is used to measure body dissatisfaction among participants (e.g., "Have you felt so bad about your shape that you have cried?"). This short 8-question version is derived from the original 34-item BSQ, and it is shown to be reliable and valid [42]. We use the Swedish BSQ-8C which has been found to exhibit high internal consistency (α=0.94) as well as excellent test-retest properties [43].

Depression Anxiety and Stress Scale (DASS-21)

Mental health problems are measured by the Depression Anxiety and Stress Scale (DASS-21) [44]. The 21 items consider the week prior to answering the survey and observe symptoms related to depression, anxiety, and stress (e.g., depression: "I felt that I had nothing to look forward to"; anxiety: "I was aware of dryness of my mouth"; stress: "I found it difficult to

For peer review only

relax”). The cut-off scores group participants into groups showing Normal, Mild, Moderate, Severe, or Extremely Severe symptoms. The exact cut-off values differ in each of the three categories. It is the most widely used version of the survey and boasts test-retest and an internal consistency of α=0.81-0.96 [44, 45].

Pittsburgh Sleep Quality Index (PSQI)

Sleep quality is measured by the Pittsburgh Sleep Quality Index (PSQI) [46] which includes 19 items and evaluates seven subcategories: Subjective sleep quality, sleep latency, sleep duration, habitual sleep efficiency, sleep disturbances, use of sleeping medications, and daytime dysfunction (e.g., “ During the past month, how often have you had trouble sleeping because you… wake up in the middle of the night or early morning”) . A Global Score is comprised of these categories on a scale of 0-21, where “0” indicates no sleep difficulty and “21” indicates severe difficulties in all areas. The PSQI has shown adequate internal consistency, reliability and construct validity. Cronbach’s alphas have been reported to 0.80 across groups and correlations between global and component scores have been shown to be moderate to high [46].

Symptoms of RED-S will be measured by Edlund Health Q, a 15-item Swedish survey focusing on menstrual cycles, injury, and eating habits.

Physical activity and Sedentary Behavior are measured by two indicator questions established by the Swedish National Board of Health and Welfare (Socialstyrelsen) [47]. The first item is regarding the amount of high intensity activity measured in time per week, such as cardiovascular training. The second item asks the amount of low intensity activity measured in time per week, such as walking or gardening. These two questions are only answered by the control group to establish eligibility among the participants.

DEXA will be used for measuring bone mineral density [48]. This noninvasive imaging technology uses a small amount of ionizing radiation to quantify the amount of bone, fat, and lean tissue, granting the examiners the opportunity to assess the risk of fracture as well as form an overall perception of the bone’s objective health and nutritional status.

The web-based questionnaire first introduces the purpose, aims, and ethics of CLIMB, emphasizing the anonymity of the participant. If the participant is <15 years of age, consent from both guardians is required. The questionnaire will be answered by all participants at baseline (rock-climbers and controls), but the follow up questionnaires will only be completed by the climbing group. This is to facilitate future data collection and analysis, as the control group data will only be analyzed at baseline as part of a cross-sectional study. The follow up measurements are therefore only required from the rock-climbers in order to provide longitudinal patterns. DEXA will only be performed on rock-climbers at baseline.

Statistical methods

Sample size

No previous data on injury incidence based on overuse injuries exist for rock climbers. Based on previous retrospective data from rock climbers [49], using a power of 80%, a significance level of 5%, and an expected relative risk of 1.5 for the primary outcome, we calculated that approximately 55 participants was needed in each group (rock climbers and control group).

For peer review only - http://bmjopen.bmj.com/site/about/guidelines.xhtml

Main exposure variables

Disordered eating behavior, body image, overuse injuries/injuries, indicators of low energy availability, mental health problems, compulsive training, perfectionism, sleep quality, sedentary behavior, and bone density.

Outcome

The outcome eating disorder, ED, will be measured with the EDE-Q. The EDE-Q consist of four subscales, restrained eating, eating concern, shape concern and weight concern addressing core dimensions of eating disorders. A score of ≥ 4 is indicative of an eating disorder, and for the present study a score of ≥ 2.5 will be used as an indication of sub-clinical eating disturbances. Thus, higher scores are indicative of more severe symptoms of eating disorder pathology.

Our *primary outcomes* are eating disorders symptoms measured with Eating Disorders Examination Questionnaire and overuse injuries measures with OSTRC-O. Our *secondary outcomes* are bone density (DEXA), symptoms of RED-S (Edlund Health Q), mental health problems (Depression, Anxiety and Stress Scale), sleep quality (Pittsburgh Sleep Quality Index), perfectionism (FMPS), compulsive exercise (CET), and body dissatisfaction (BSQ-8).

Data analysis plan

Multinominal logistic regression analyses will be used to determine the associations between each of the exposures and trajectories of outcomes. The associations as ORs and 95% CIs will be reported. Bivariate models to measure the crude associations between the exposures and trajectories will be built.

The differences between the rock-climbers and controls regarding the above-mentioned outcome measure *eating disorder*, ED, will be analyzed using multivariate analysis of variance MANOVA. Both within- and between groups analysis will be conducted. Further analyses among rock-climbers with high ED vs low ED will be compared in relation to bone density, injuries, mental health, and symptoms of RED-S. The multivariate analysis of variance MANOVA will be used for that purpose. A one-way repeated measures ANOVA will be used to identify changes over the two-year span in the two groups of rock-climbers with high ED vs. low ED. In cases where overall significant changes are detected, post-hoc analysis will be applied to specify where the between group differences exist.

Structural equation modelling (SEM) will be used at a later stage to investigate if the variables body image, injuries, mental illness, sports environment, bone density (from baseline), and eating disorders can predict injuries.

Ethics and dissemination

All participants will provide informed consent to participate in CLIMB after they had been informed about the purpose and procedure of the study and that it has been approved by the Swedish Ethical Review Authority (reference number: 2021-05557-01).

The dissemination plan is to present our results to the climbing and sport communities, in peer-reviewed scientific journals, at congresses, and to stakeholders with influence in the development of climbing environments.

For peer review only

Study status

The manuscript reports the protocol (ClinicalTrials.gov NCT05587270) for an ongoing study for which participants is currently ongoing. Data collection has been initiated and baseline analyses are planned to commence during 2023.

Data Availability Statement
Data sharing is not applicable to this article as no new data were created or analyzed in this study.

DISCUSSION

Climbing is a weight sensitive sport where athletes generally benefit from a lean body shape and low weight [6]- a risk factor for eating disturbances. There are very few studies regarding eating disturbances among rock-climbers. A study by Joubert et. al., examining disordered eating among international sport lead rock climbers, displayed that 6.3 % of male and 16.5 % of female climbers had disordered eating [30]. Similarly, a study examining amenorrhea among female competitive climbers, showed that 15.8% of female climbers had amenorrhea. The IFSC recently released a statement to counteract this development of the sport. Due to the overrepresentation of eating disturbances reported in other lean sports and the poor treatment prognosis, early detection and prevention of eating disturbances and energy deficiency is essential, especially considering the growing popularity of the sport.

There are several major strengths of CLIMB. The first is the use of longitudinal data within several domains related to disturbed eating and mental health issues by using validated questionnaires, reducing misclassification of outcomes. The few studies published at this time, on this topic, do not consider multiple mental health parameters that CLIMB intends to address, such as perfectionism, sleep habits, body dissatisfaction, and compulsive exercise. It is the first study on rock-climbers which also includes a control group, providing vital context on disease prevalence. CLIMB will be representative of the advanced climbing community at large in Sweden as it includes a wide age span of both genders who climb at advanced and elite levels around Sweden, markedly decreasing the risk of selection bias. Additionally, this large sample of climbers contributes to ensuring external validity. The use of DEXA complements the participant's self-report data with objective measurements on the effects that Relative Energy Deficiency is associated with, establishing a point of action and initiative to prevent and treat afflicted athletes.

One limitation of our study is that we are using a non-random convenience sample that may not be representative to advanced rock-rock-climbers in an international perspective. We have aimed to include the whole advanced level rock-climbing community in Sweden aged 15 years and older, which is the rational for how the participants were recruited. According to the Swedish Climbing Association, there were 15409 climbers affiliated to a climbing club in 2022. It is unknown, however, how many of climbers are of an advanced or higher level, and thus eligible for participating in the study. Another potential limitation is the use of self-report questionnaires only which yield subjective data. Although the authors will encourage all participants to fill out all surveys using communication strategies at baseline and throughout the follow-up surveys, there is an undeniable risk of increased drop-out rates with each passing survey.

For peer review only

The climbing-concerned scientific community is at the beginning of forming a paradigm around this subject. The many facets of this study make CLIMB invaluable in gaining a holistic view on each participant as well as on group levels, which ultimately contributes to developing and implementing evidence-based interventions around the globe.

Authors' contributions

Dr. Sansone is the project director and Dr. Edlund the co-project director, and they have together with the senior researchers Dr. Hedelin and Dr. Tranaeus contributed to the planning of the study as well as the conception and design. They, together with PhD students Dr. Nigicser and Dr. Identeg have contributed to writing this study protocol. Mr. Forsberg is a research assistant in the project. They have contributed to the planning, conception and design of the study including planning and executing the digital survey. They have all contributed to the recruitment of the participants and administered the work around setting up the study. Dr. Tranaeus has also performed the DEXA measures. All authors have contributed to the writing process with input, feedback and critically revising this study protocol. The manuscript was approved by all authors.

Funding statement: This research received no specific grant from any funding agency in the public, commercial or not-for-profit sectors.

Competing interest statement: The authors declare that they have no competing interests.

Word count: 4045

References:

1. Sundgot-Borgen, J., et al., *How to minimise the health risks to athletes who compete in weight-sensitive sports review and position statement on behalf of the Ad Hoc Research Working Group on Body Composition, Health and Performance, under the auspices of the IOC Medical Commission.* Br J Sports Med, 2013. **47**(16): p. 1012-22.
2. Giel, K.E., et al., *Eating disorder pathology in elite adolescent athletes.* Int J Eat Disord, 2016. **49**(6): p. 553-62.
3. Papageorgiou, M., et al., *Reduced energy availability: implications for bone health in physically active populations.* Eur J Nutr, 2018. **57**(3): p. 847-859.
4. Herpertz-Dahlmann, B., *Adolescent eating disorders: update on definitions, symptomatology, epidemiology, and comorbidity.* Child Adolesc Psychiatr Clin N Am, 2015. **24**(1): p. 177-96.
5. Holtkamp, K., J. Hebebrand, and B. Herpertz-Dahlmann, *The contribution of anxiety and food restriction on physical activity levels in acute anorexia nervosa.* Int J Eat Disord, 2004. **36**(2): p. 163-71.
6. Hulley, A.J. and A.J. Hill, *Eating disorders and health in elite women distance runners.* Int J Eat Disord, 2001. **30**(3): p. 312-7.
7. Joy, E., et al., *2014 female athlete triad coalition consensus statement on treatment and return to play of the female athlete triad.* Curr Sports Med Rep, 2014. **13**(4): p. 219-32.

For peer review only

8. Byrne, S. and N. McLean, *Elite athletes: effects of the pressure to be thin.* J Sci Med Sport, 2002. **5**(2): p. 80-94.
9. Werner, A., et al., *Weight-control behaviour and weight-concerns in young elite athletes - a systematic review.* J Eat Disord, 2013. **1**: p. 18.
10. Ravaldi, C., et al., *Eating disorders and body image disturbances among ballet dancers, gymnasium users and body builders.* Psychopathology, 2003. **36**(5): p. 247-54.
11. Mountjoy, M., et al., *The IOC consensus statement: beyond the Female Athlete Triad--Relative Energy Deficiency in Sport (RED-S).* Br J Sports Med, 2014. **48**(7): p. 491-7.
12. Smink, F.R., D. van Hoeken, and H.W. Hoek, *Epidemiology of eating disorders: incidence, prevalence and mortality rates.* Curr Psychiatry Rep, 2012. **14**(4): p. 406-14.
13. Lutter, C., et al., *Sport climbing: medical considerations for this new Olympic discipline.* Br J Sports Med, 2017. **51**(1): p. 2-3.
14. Papageorgiou, M., et al., *Effects of reduced energy availability on bone metabolism in women and men.* Bone, 2017. **105**: p. 191-199.
15. Joubert, L., et al., *Prevalence of amenorrhea in elite female competitive climbers.* Front Sports Act Living, 2022. **4**: p. 895588.
16. Forsén Mantilla, E., et al., *Impulsivity and compulsivity as parallel mediators of emotion dysregulation in eating-related addictive-like behaviors, alcohol use, and compulsive exercise.* Brain Behav, 2022. **12**(1): p. e2458.
17. Godier, L.R. and R.J. Park, *A novel measure of compulsive food restriction in anorexia nervosa: validation of the Self-Starvation Scale (SS).* Eat Behav, 2015. **17**: p. 10-3.
18. Bunio, L.K., *The nuances of emotion regulation difficulties and mindfulness in food addiction*, J.A. Battles, Loverich, Tamara M., Editor. 2021, Addiction Research & Theory. p. 11-17.
19. Edlund, K., et al., *Body image and compulsive exercise: are there associations with depression among university students?* Eat Weight Disord, 2022. **27**(7): p. 2397-2405.
20. Dyrbye, L.N., M.R. Thomas, and T.D. Shanafelt, *Systematic review of depression, anxiety, and other indicators of psychological distress among U.S. and Canadian medical students.* Acad Med, 2006. **81**(4): p. 354-73.
21. Torstveit, M.K., J.H. Rosenvinge, and J. Sundgot-Borgen, *Prevalence of eating disorders and the predictive power of risk models in female elite athletes: a controlled study.* Scand J Med Sci Sports, 2008. **18**(1): p. 108-18.
22. Stice, E. and H.E. Shaw, *Role of body dissatisfaction in the onset and maintenance of eating pathology: a synthesis of research findings.* J Psychosom Res, 2002. **53**(5): p. 985-93.
23. Sundgot-Borgen, J., *Risk and trigger factors for the development of eating disorders in female elite athletes.* Med Sci Sports Exerc, 1994. **26**(4): p. 414-9.
24. Sundgot-Borgen, J. and M.K. Torstveit, *Aspects of disordered eating continuum in elite high-intensity sports.* Scand J Med Sci Sports, 2010. **20 Suppl 2**: p. 112-21.
25. Sundgot-Borgen, J. and M.K. Torstveit, *Prevalence of eating disorders in elite athletes is higher than in the general population.* Clin J Sport Med, 2004. **14**(1): p. 25-32.

For peer review only

26. Hargreaves, D.A. and M. Tiggemann, *Idealized media images and adolescent body image: "comparing" boys and girls.* Body Image, 2004. **1**(4): p. 351-61.

27. Joy, E., A. Kussman, and A. Nattiv, *2016 update on eating disorders in athletes: A comprehensive narrative review with a focus on clinical assessment and management.* Br J Sports Med, 2016. **50**(3): p. 154-62.

28. Bar, R.J., S.E. Cassin, and M.M. Dionne, *Eating disorder prevention initiatives for athletes: A review.* Eur J Sport Sci, 2016. **16**(3): p. 325-35.

29. Draper, N.e.a., *Comparative grading scales, statistical analyses, climber descriptors and ability grouping: International Rock Climbing Research Association position statement*. 2016, Taylor and Francis: Sports Technology. p. 88-94.

30. Joubert, L.M., G.B. Gonzalez, and A.J. Larson, *Prevalence of Disordered Eating Among International Sport Lead Rock Climbers.* Front Sports Act Living, 2020. **2**: p. 86.

31. Strand, M., *Attitudes towards disordered eating in the rock climbing community: a digital ethnography.* J Eat Disord, 2022. **10**(1): p. 96.

32. Mountjoy, M., et al., *International Olympic Committee (IOC) Consensus Statement on Relative Energy Deficiency in Sport (RED-S): 2018 Update.* Int J Sport Nutr Exerc Metab, 2018. **28**(4): p. 316-331.

33. Fairburn, C.G. and S.J. Beglin, *Assessment of eating disorders: interview or self-report questionnaire?* Int J Eat Disord, 1994. **16**(4): p. 363-70.

34. Carter, J.C., D.A. Stewart, and C.G. Fairburn, *Eating disorder examination questionnaire: norms for young adolescent girls.* Behav Res Ther, 2001. **39**(5): p. 625-32.

35. Clarsen, B., G. Myklebust, and R. Bahr, *Development and validation of a new method for the registration of overuse injuries in sports injury epidemiology: the Oslo Sports Trauma Research Centre (OSTRC) overuse injury questionnaire.* Br J Sports Med, 2013. **47**(8): p. 495-502.

36. Frost, R., et al., *The dimensions of perfectionism.* 1990: Cognit Ther Res. p. 49-68.

37. Suddarth, B.H. and R.B. & Slaney, *An investigation of the dimensions of perfectionism in college students*. 2001: *Measurement and Evaluation in Counseling and Development*. p. 157–165.

38. Bußenius, L. and S. Harendza, *The relationship between perfectionism and symptoms of depression in medical school applicants.* BMC Med Educ, 2019. **19**(1): p. 370.

39. Taranis, L., S. Touyz, and C. Meyer, *Disordered eating and exercise: development and preliminary validation of the compulsive exercise test (CET).* Eur Eat Disord Rev, 2011. **19**(3): p. 256-68.

40. Plateau, C., *Use of the compulsive exercise test with athletes: Norms and links with eating psychopathology.*, V. Shanmugam, Duckham RL., Editor. 2014, Journal of Applied Sport Psychology. p. 287-301.

41. Welch, E., M. Lagerström, and A. Ghaderi, *Body shape questionnaire: psychometric properties of the short version (BSQ-8C) and norms from the general Swedish population.* Body Image, 2012. **9**(4): p. 547-50.

42. Evans, C. and B. Dolan, *Body Shape Questionnaire: derivation of shortened "alternate forms".* Int J Eat Disord, 1993. **13**(3): p. 315-21.

For peer review only - http://bmjopen.bmj.com/site/about/guidelines.xhtml

43. Edlund, K., et al., *Sustainable UNiversity Life (SUN) study: protocol for a prospective cohort study of modifiable risk and prognostic factors for mental health problems and musculoskeletal pain among university students.* BMJ Open, 2022. **12**(4): p. e056489.

44. Lovibond, P.F. and S.H. Lovibond, *The structure of negative emotional states: comparison of the Depression Anxiety Stress Scales (DASS) with the Beck Depression and Anxiety Inventories.* Behav Res Ther, 1995. **33**(3): p. 335-43.

45. Alfonsson, S., E. Wallin, and P. Maathz, *Factor structure and validity of the Depression, Anxiety and Stress Scale-21 in Swedish translation.* J Psychiatr Ment Health Nurs, 2017. **24**(2-3): p. 154-162.

46. Carpenter, J.S. and M.A. Andrykowski, *Psychometric evaluation of the Pittsburgh Sleep Quality Index.* J Psychosom Res, 1998. **45**(1): p. 5-13.

47. Socialstyrelsen, *Nationella riktlinjer för sjukdomsförebyggande metoder: Tobaksbruk, riskbruk av alkohol, otillräcklig fysisk aktivitet och ohälsosamma matvanor. Stöd för styrning och ledning.* 2011: Stockholm. p. Indikatorer (extern bilaga), p. 14-23.

48. Kim, H.S., et al., *Bone mineral density assessment for research purpose using dual energy X-ray absorptiometry.* Osteoporos Sarcopenia, 2018. **4**(3): p. 79-85.

49. Jones, G., D. Llewellyn, and M.I. Johnson, *Previous injury as a risk factor for reinjury in rock climbing: a secondary analysis of data from a retrospective cross-sectional cohort survey of active rock climbers.* BMJ Open Sport Exerc Med, 2015. **1**(1).

For peer review only

STROBE Statement—Checklist of items that should be included in reports of ***cohort studies***

	Item No	Recommendation	Page No
**Title and abstract**	1	(*a*) Indicate the study's design with a commonly used term in the title or the abstract<br>(*b*) Provide in the abstract an informative and balanced summary of what was done and what was found	1-2
**Introduction**			
Background/rationale	2	Explain the scientific background and rationale for the investigation being reported	2-4
Objectives	3	State specific objectives, including any prespecified hypotheses	4
**Methods**			
Study design	4	Present key elements of study design early in the paper	4
Setting	5	Describe the setting, locations, and relevant dates, including periods of recruitment, exposure, follow-up, and data collection	4-5
Participants	6	(*a*) Give the eligibility criteria, and the sources and methods of selection of participants. Describe methods of follow-up<br>(*b*) For matched studies, give matching criteria and number of exposed and unexposed	4-5
Variables	7	Clearly define all outcomes, exposures, predictors, potential confounders, and effect modifiers. Give diagnostic criteria, if applicable	4-8
Data sources/ measurement	8*	For each variable of interest, give sources of data and details of methods of assessment (measurement). Describe comparability of assessment methods if there is more than one group	4-8
Bias	9	Describe any efforts to address potential sources of bias	n/a
Study size	10	Explain how the study size was arrived at	7
Quantitative variables	11	Explain how quantitative variables were handled in the analyses. If applicable, describe which groupings were chosen and why	n/a
Statistical methods	12	(*a*) Describe all statistical methods, including those used to control for confounding<br>(*b*) Describe any methods used to examine subgroups and interactions<br>(*c*) Explain how missing data were addressed<br>(*d*) If applicable, explain how loss to follow-up was addressed<br>(*e*) Describe any sensitivity analyses	8
**Results**			
Participants	13*	(a) Report numbers of individuals at each stage of study—eg numbers potentially eligible, examined for eligibility, confirmed eligible, included in the study, completing follow-up, and analysed<br>(b) Give reasons for non-participation at each stage<br>(c) Consider use of a flow diagram	n/a
Descriptive data	14*	(a) Give characteristics of study participants (eg demographic, clinical, social) and information on exposures and potential confounders<br>(b) Indicate number of participants with missing data for each variable of interest<br>(c) Summarise follow-up time (eg, average and total amount)	n/a
Outcome data	15*	Report numbers of outcome events or summary measures over time	n/a

For peer review only

For peer review only - http://bmjopen.bmj.com/site/about/guidelines.xhtml

Main results	16	(*a*) Give unadjusted estimates and, if applicable, confounder-adjusted estimates and their precision (eg, 95% confidence interval). Make clear which confounders were adjusted for and why they were included<br>(*b*) Report category boundaries when continuous variables were categorized<br>(*c*) If relevant, consider translating estimates of relative risk into absolute risk for a meaningful time period	n/a
Other analyses	17	Report other analyses done—eg analyses of subgroups and interactions, and sensitivity analyses	n/a
**Discussion**			
Key results	18	Summarise key results with reference to study objectives	n/a
Limitations	19	Discuss limitations of the study, taking into account sources of potential bias or imprecision. Discuss both direction and magnitude of any potential bias	
Interpretation	20	Give a cautious overall interpretation of results considering objectives, limitations, multiplicity of analyses, results from similar studies, and other relevant evidence	n/a
Generalisability	21	Discuss the generalisability (external validity) of the study results	n/a
**Other information**			
Funding	22	Give the source of funding and the role of the funders for the present study and, if applicable, for the original study on which the present article is based	12

*Give information separately for exposed and unexposed groups.

Note: An Explanation and Elaboration article discusses each checklist item and gives methodological background and published examples of transparent reporting. The STROBE checklist is best used in conjunction with this article (freely available on the Web sites of PLoS Medicine at http://www.plosmedicine.org/, Annals of Internal Medicine at http://www.annals.org/, and Epidemiology at http://www.epidem.com/). Information on the STROBE Initiative is available at http://www.strobe-statement.org.

For peer review only
